# Protein-protein interaction prediction using bidirectional GRUs with explicit ensemble

**DOI:** 10.1371/journal.pone.0326960

**Published:** 2025-07-02

**Authors:** Qiuhong Lan, Zhongtuan Zheng, Zhen Tang, Xuehua Qiu, Zhixiang Yin

**Affiliations:** 1 School of Mathematics, Physics and Statistics, Shanghai University of Engineering Science, Shanghai, China; 2 Institute for Frontier Medical Technology, Shanghai Frontiers Science Research Center for Druggability of Cardiovascular Noncoding RNA, Center of Intelligent Computing and Applied Statistics, Shanghai University of Engineering Science, Shanghai, China; National University of Singapore, SINGAPORE

## Abstract

Protein-protein interactions is essential for cellular processes in all organisms. The accurate in-silico identification of these interactions is a significant area of research in biology-related fields, which is crucial for protein function prediction and drug design. Protein sequence data serves as the primary source for computational protein prediction. However, existing models for predicting protein-protein interactions based on sequence information typically consider only a limited set of physicochemical properties of amino acids. Consequently, they fail to comprehensively characterize protein sequence information, resulting in models that perform well within the species for which they were trained but poorly in cross-species environments. Unlike previous models, this paper combines the SVHEHS descriptor with various feature coding techniques to characterize protein sequences more comprehensively. The model employs explicit integration of bidirectional gated recurrent units to fuse multi-information. The final model achieves prediction accuracies of 96.47% and 97.79% on the *H. pylori* and *S. cerevisiae* datasets, respectively, outperforming most current models reported in the literature. In particular, the experimental results indicate that the model exhibits strong generalizability across various species datasets, suggesting it can serve as a valuable reference for investigating protein interaction networks in different species.

## Introduction

Analyzing protein-protein interactions (PPIs) is the basis for understanding biological life activities [1]. Effective prediction of PPIs can help in key protein identification [2], protein complex mining, and disease-causing gene screening [3], and can also provide a reference for new drug development of drugs targeting protein interactions [4]. Early prediction of PPIs relies on traditional experimental methods, which mainly contain tandem affinity purification (TAP) [5], immunoprecipitation [6], yeast two-hybrid technique [7], etc. These methods have greatly contributed to the development of proteomics, but are extremely time-consuming and labor-intensive due to the large number of experiments required for repeated validation [8]. At the same time, due to limited technology and other reasons, experiments are more likely to produce a large number of false positives and false negatives. The PPIs validated so far with biochemical experiments are still the tip of the iceberg of the entire PPI dataset [9]. The fortunate thing is that with the rapid progress of computational technology, computational methods became a powerful complement to the means of predicting PPIs, and gradually became the mainstream methods. The application of machine learning techniques to study PPIs began in 2001, primarily through the independent efforts of research groups led by Bock and Gough, Sprinzak and Margalit, and Zhou and Shan [10]. Its groundbreaking research results signify the beginning of a new era in the study of PPIs through the application of machine learning techniques. For instance, Shen *et al*. [11] categorized 20 amino acids into seven classes based on their dipole and side chain volume. They represented protein sequences by quantifying the distribution of triplets within these sequences and subsequently inputted the features into a support vector machine, achieving an accuracy of 83.9% in predicting interactions within a human protein dataset. PIPR [12] is an end-to-end framework that integrates deep residual recurrent convolutional neural networks within a Siamese architecture to effectively capture the interactions between pairs of protein sequences. Experimental results demonstrate that PIPR outperforms the state-of-the-art systems available at the time in predicting binary PPIs.

For predicting PPIs, the key to successful outcomes lies in two main areas. The first crucial step is to develop high-quality feature sets. Existing machine learning models can be broadly classified into three categories based on the types of features utilized: sequence-based, structure-based, and hybrid methods that combine both approaches. As researchers contribute to the exponential growth of protein sequence databases through macro-genome sampling, a substantial amount of essential data for studying PPIs based on protein sequence information becomes available [13]. Furthermore, protein sequence information significantly influences the higher-level structural and biological properties of proteins. Consequently, we can infer structure and function from patterns within the sequences of a protein family. This understanding has also inspired new research into evolutionary-scale language models, emphasizing the application of large-scale language models to directly deduce structure from primary protein sequences [13]. Hu *et al*. [14] also note that the knowledge derived from protein sequences is sufficient for estimating the likelihood of interactions between pairs of proteins. Therefore, sequence data have been widely utilized by researchers due to their greater availability and reduced need for background knowledge in data preprocessing compared to structural data. To this day, sequence data remains the primary source of information for predicting PPIs [15]. After completing feature extraction, the next critical step in predicting PPIs is selecting an appropriate classification model to ensure accurate results. PPIs prediction is essentially a binary classification problem [16]. While a large number of feature coding techniques have been developed, traditional machine learning models applicable to binary classification problems have been heavily applied by scholars to the prediction of PPIs [17–19]. In recent years, deep learning has attracted considerable attention from researchers across various fields, including text analysis, speech recognition, and image processing, due to its effectiveness in unsupervised feature learning. Additionally, numerous initiatives have emerged to apply deep learning techniques for predicting PPIs [20–22]. Particularly noteworthy is the extensive use of pre-trained protein language models for feature extraction in predicting protein interactions, which has led to a series of breakthroughs in predictive performance [23–25]. Despite significant advancements in PPIs prediction, most existing models exhibit limited generalizability. These models tend to perform well on the training set but show reduced performance on the test set. This decline can be attributed, in part, to a lack of comprehensive and sufficiently distinct information regarding the characterized protein sequences [23].

Considering the potential to improve the construction of protein feature sets, this paper proposes a model for predicting PPIs using bidirectional gated recurrent units (BiGRUs) in combination with an explicit ensemble approach. Firstly, based on an extensive study of techniques for obtaining feature sets from protein sequence information, and considering the objectives of ease of implementation and a low information repetition rate, this paper ultimately selects six feature coding techniques: pseudo amino acid composition (PseAAC), autocorrelation descriptor (AD), autocovariance (AC), conjoint triad (CT), local descriptor (LD), and multivariate mutual information (MMI). These techniques will be employed to convert protein sequences into feature vectors. They gather protein sequence information from various sources, including compositional data, sequential information, and both long-range and short-range interactions, to enhance prediction accuracy. Meanwhile, to improve the comprehensive characterization of protein sequence information, this paper substitutes the original data used in the first three techniques with the SVHEHS descriptor proposed by Peng *et al*. [26]. This descriptor is a 20 × 13-dimensional representation derived from 457 physicochemical properties of amino acids, following principal component analysis. Since the latter three techniques classify amino acids into categories based on dipole and side chain volume values, there is insufficient scientific justification for applying a similar classification to the SVHEHS descriptor. Consequently, these three techniques were not employed for information replacement. Secondly, the feature vectors obtained from each feature coding technique are input into BiGRUs for data reduction. The output dimension of the GRU layer is determined by the dimension of the input feature vectors. Finally, a subset of the six classes of optimal features are concatenated by protein pairs and fed into the LightGBM classifier for five-fold cross-validation. The results show that the prediction accuracy of the model on the *H. pylori* and *S. cerevisiae* datasets are 96.47% and 97.79%, respectively, which has improved accuracy compared to most current literature models. In addition, the model achieves satisfactory performance on the datasets of *Disease-specific*, *One-core network*, and *Wnt-related pathway*, confirming its potential to serve as a valuable resource for signaling pathway research, disease-causing gene identification, and human disease prevention. This model is filtered and determined through a layer-by-layer meritocracy. Our contributions can be summarized as follows: (1) The introduction of the SVHEHS descriptor as the information for the three feature coding techniques, namely, PseAAC, AD, and AC, provides a more comprehensive characterization of the protein sequences than the use of the original information. The multi-information fusion approach further improves the quality of the feature set. (2) Data dimensionality reduction is achieved by the BiGRU, where the output dimension of the GRU layer is determined based on the dimension of the input feature vector and according to a certain computational law. (3) The BiGRU extracts a more comprehensive subset of optimal features than the unidirectional GRU, and the model is explicitly integrated in a way that can improve the performance of a single BiGRU.

## Materials and methods

### Datasets

This paper involves nine public PPI datasets. The first dataset: *Helicobacter pylori* (*H. pylori*), was constructed by Martin *et al*. [27], which contains 1,458 positive and 1,458 negative samples. The second dataset: *Saccharomyces cerevisiae* (*S. cerevisiae*), was constructed by Guo *et al*. [28] and includes 5,594 interacting protein pairs and 5,594 non-interacting protein pairs. All of the protein sequences are greater than or equal to 50 in length and the similarity between the sequences is less than 40%. The model is evaluated using five-fold cross-validation on the *H. pylori* and *S. cerevisiae* datasets. The prediction results are then utilized to assess the model’s overall performance. To evaluate the generalization capability of the model, we assess the model trained on the *S. cerevisiae* dataset using four independent protein-protein interaction datasets and three protein-protein interaction network datasets. The first type of test set: *Caenorhabditis elegans* (*C. elegans*, 4,013 pairs of proteins), *Escherichia coli* (*E. coli*, 6,954 pairs of proteins), *Homo sapiens* (*H. sapiens*, 1,412 pairs of proteins) and *Mus musculus* (*M. musculus*, 313 pairs of proteins) [29]. The second type of test set: *Disease-specific* (108 pairs of proteins) [30], *One-core network* (16 pairs of proteins) [31], and *Wnt-related pathway* (96 pairs of proteins) [32].

### Feature coding techniques

#### Feature coding techniques based on the SVHEHS descriptor.

The SVHEHS descriptor is a 20 × 13-dimensional amino acid structure descriptor obtained by classifying 457 physicochemical property parameters of 20 natural amino acids collected from the AA index database [33] based on hydrophobic properties, electronic properties, hydrogen bonds contributions, and steric properties. This classification was done by Peng *et al*. [26], who then performed principal component analysis for each parameter. For more information, refer to S1 File Sect 1, S1 Table. The experimental results [26,34,35] indicated that models developed using this descriptor for sequence characterization demonstrated good prediction accuracy and fitting ability. This, to some extent, highlighted the validity and comprehensiveness of the sequence characterization method.

**Pseudo amino acid composition (PseAAC):** Chou [36] first proposed the pseudo amino acid composition (PseAAC) feature coding technique in 2001, which can simultaneously characterize the compositional and sequential information of amino acids involving hydrophobicity, hydrophilicity, and side chain quality. In this paper, the three amino acid physicochemical properties involved in the original formulas are replaced with the SVHEHS descriptor. The computational formulas are in the S1 File Sect 2.1.1.

**Autocorrelation descriptor (AD):** The autocorrelation descriptor (AD) [37] feature coding technique includes the Moreau-Broto autocorrelation descriptor, Moran autocorrelation descriptor, and Geary autocorrelation descriptor. This technique considers both the long-range and short-range effects of protein sequences, incorporating seven physicochemical properties of amino acids. In this paper, the seven physicochemical properties of amino acids in the original formulas are substituted with the SVHEHS descriptor. The computational formulas are elaborated in the S1 File Sect 2.1.2.

**Auto covariance (AC):** The auto covariance (AC) feature coding technique was proposed by Guo *et al*. [28]. This technique calculates the self-covariance of the same descriptor for two amino acids in a protein sequence. It involves the physicochemical properties and remote interactions of the amino acids. The physicochemical properties include hydrophobicity, hydrophilicity, amino acid side-chain volume, polarity, polarizability, solvent-accessible surface area of the amino acid side-chain, and the net charge index. In this paper, the physicochemical properties in the original formulas are replaced with the SVHEHS descriptor. The calculations are detailed in the S1 File Sect 2.1.3.

#### Feature coding techniques based on dipole and side chain volume.

The interactions between amino acids can be dominated by the dipole and side chain volume of the amino acids. These interactions can be classified into seven categories based on the variation in the degree to which these two properties are present in different amino acids [29,38], as outlined in S1 File Sect 2.2, S2 Table.

**Conjoint triad (CT):** The conjoint triad (CT) [11] feature coding technique is based on the classification of amino acids into seven classes according to dipole and side chain volume. This technique considers the interactions between neighboring amino acids and treats three adjacent amino acid molecules as a whole, defined as a triplet. It calculates the frequency of individual triplets in each protein sequence, normalizes it by subtracting the minimum value and then dividing by the maximum. Detailed calculations can be found in the S1 File Sect 2.2.1.

**Local descriptor (LD):** The local descriptor (LD) [39] feature coding technique considers that discontinuous regions in a protein sequence can be spatially close due to protein folding. It is believed that interactions between continuous and discontinuous sequences of proteins may also exist. The technique initially categorizes amino acids into seven groups based on their dipole and side chain volume. Subsequently, it divides each protein sequence into 10 regions. For each subsequence, three local descriptors - composition (C), transition (T), and distribution (D) - are calculated individually. Specific details are in the S1 File Sect 2.2.2.

**Multivariate mutual information (MMI):** The multivariate mutual information (MMI) feature coding technique was proposed by Ding *et al*. [40]. This technique applies the concepts of mutual information and entropy methods to protein sequences. Based on the classification of amino acids into seven categories according to dipole and side chain volume, the number of 3-gram and 2-gram features is calculated without considering the order of occurrence of amino acids. The 3-gram features consist of triplets of three adjacent amino acid molecules on a protein sequence, while the 2-gram features consist of dipeptides of two adjacent amino acid molecules on a protein sequence. The mutual information of 3-gram features and 2-gram features, along with the frequency of occurrence of each type of amino acid in the protein sequence, are used as components of the multivariate mutual information feature coding technique. The detailed calculations are in the S1 File Sect 2.2.3.

### Model construction

#### Overall framework of the model.

**Fig 1:**
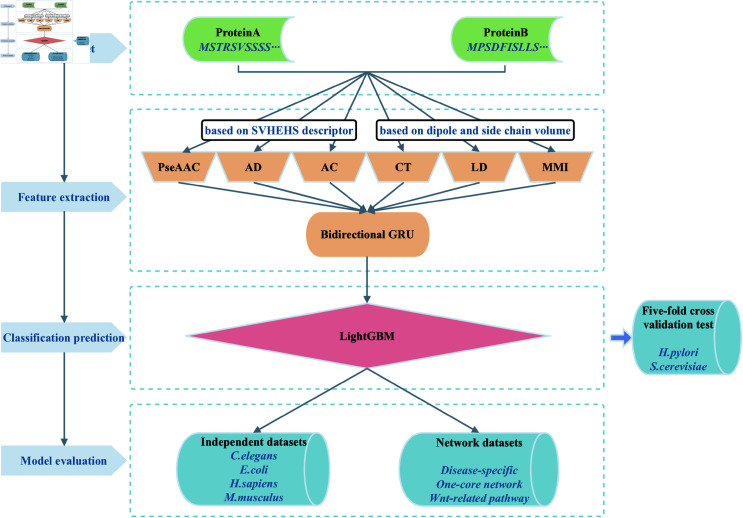
Framework of the model.

In this paper, we propose a model for predicting PPIs using BiGRUs with an explicit ensemble approach. The framework of the model is shown in Fig 1. This framework is implemented using MATLAB 2019b and Python 3.8.

The inputs to the model are protein sequence pairs. Six feature coding techniques transform protein sequences into digital representations that can be processed by the model. The BiGRU (refer to S1 File Sect 3.1 for a detailed description of the BiGRU) achieves data dimensionality reduction and access to high-quality datasets. Adam is selected as the optimizer for the BiGRU. The learning rate is set to 0.001, the gradient clipping parameter is established at 1, the loss function used is binary cross-entropy, and the BiGRU is trained for five epochs. LightGBM (refer to S1 File Sect 3.2 for a detailed description of the LightGBM) uses the BiGRU-processed data for interaction prediction of the protein sequence pairs. The number of iterations of LightGBM is set to 500, the random seed is set to 1, and the other default parameters are used. In this study, parameter optimization of the classifier and model evaluation are performed using five-fold cross-validation. Five-fold cross-validation involves dividing the dataset into five mutually exclusive subsets of approximately equal size. In each iteration, four subsets are combined to form the training set, while the remaining subset serves as the test set. This process generates five distinct pairs of training and testing sets, enabling five separate training and testing sessions. The average result from these five test sets is then utilized as the final prediction.

The specific steps of the model for PPIs prediction are as follows.

- PPIs datasets. Enter *H. pylori*, *S. cerevisiae*, four cross-species datasets (*C. elegans*, *E. coli*, *H. sapiens*, *M. musculus*), and three disease-associated datasets (*Disease-specific*, *One-core network*, *Wnt-related pathway*).
- Feature extraction. Firstly, six feature coding techniques are used: PseAAC, AD, AC, CT, LD, and MMI. These techniques transform protein sequences into feature vectors. Parameters for the first three feature coding techniques also need to be determined. The resulting vectors are then fed into various BiGRUs based on different feature coding techniques to generate high-quality and representative datasets.
- Classification prediction. The six types of data obtained from the feature extraction module are combined and input into the LightGBM classifier to predict interactions among proteins in the training set.
- Model evaluation. The model trained using *S. cerevisiae* is tested on cross-species test sets (*C. elegans*, *E. coli*, *H. sapiens*, *M. musculus*) and disease-related test sets (*Disease-specific*, *One-core network*, *Wnt-related pathway*) to evaluate the generalization performance of the model.

**Fig 2:**
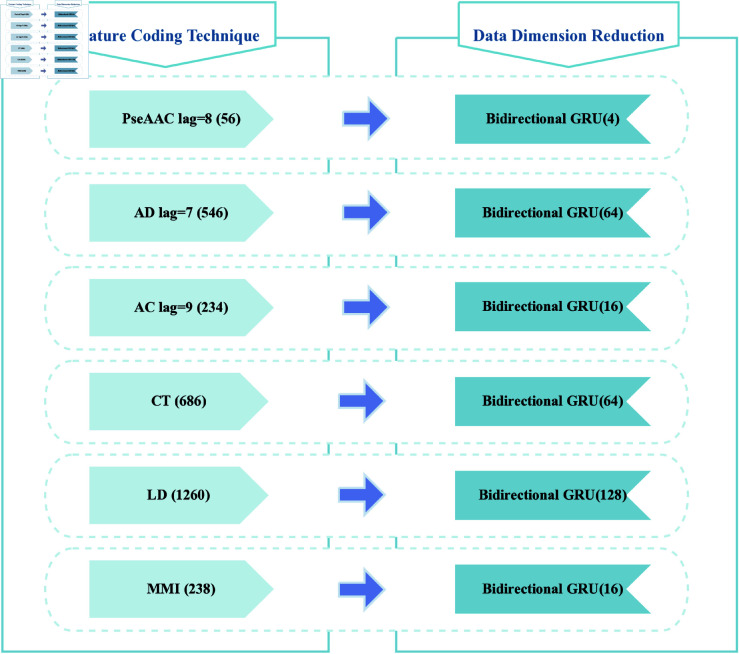
Detailed view of the feature extraction module. (Note: The number of vector dimensions of the resulting protein pairs is labeled in parentheses after each feature coding technique, and the size of the output dimension of the GRU layer in the BiGRU is labeled in parentheses after each BiGRU.)

**Fig 3:**
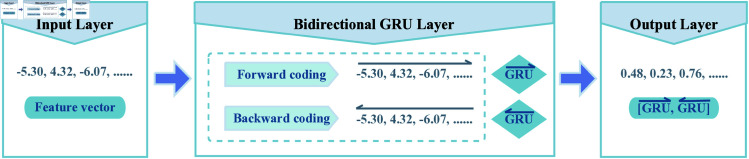
Structure of the BiGRU.

Among them, the feature extraction module can be divided into feature coding and data dimensionality reduction. The specific details are shown in Figs 2 and 3.

In this paper, we employ an explicit ensemble method to merge datasets acquired from six feature coding techniques. Specifically, each feature coding technique is linked to a BiGRU. The output dimension of the GRU layer in the BiGRU is determined by both the dimension of the input feature vectors and a specific computational rule. This is computed by taking the largest integer not greater than the *n-th* power of 2 of the dimension of the input feature vector, with *n* being the parameter to be determined. Considering that the final output data dimension of the BiGRU is twice as much as that of the unidirectional GRU, and aiming to achieve data dimensionality reduction, the size of the final power needs to be reduced by 3 from the original. For example, the dimension of the feature vectors obtained by applying the PseAAC feature coding technique is 56. The largest integer not greater than *n* powers of 2 is 32, which means *n* = 5. Therefore, 5−3=2, and the output dimension of the finalized GRU layer is 2^2^ = 4, resulting in the BiGRU output dimension being 8. The datasets passing through the six BiGRUs are merged, resulting in a 292×2=584-dimensional feature vector.

Gated recurrent unit (GRU), is a variant of the recurrent neural network (RNN) that is commonly used to process data with explicit temporal dependencies. Its architecture, which is based on recurrent neural networks, effectively captures the underlying sequential information within the feature vector. On the other hand, Abien Fred M. Agarap [41] observed that replacing the commonly used Softmax function with a Support Vector Machine (SVM) in the final output layer of the GRU resulted in the GRU-SVM model achieving higher prediction accuracy compared to the traditional GRU-Softmax model. Based on the two points mentioned above, we design a BiGRU consisting of three layers: the input layer, the bi-directional GRU layer, and the output layer, as illustrated in Fig 3. The input protein pairs for the model are transformed into feature vectors using various coding techniques. These vectors are then processed through a bidirectional GRU layer, resulting in output feature vectors commonly referred to as hidden states. It is important to note that some of the feature coding techniques employed in this paper utilize discretization methods. Specifically, there are ’pseudo-sequence’ feature vectors that are accompanied by frequencies in the generated feature vectors. However, this does not imply that these feature vectors lack potential sequential information. Take the conjoint triad (CT) feature coding technique as an example. In the CT method, the frequent occurrence of certain types of amino acids leads to a high frequency of specific triplets. This increased frequency of triplets may suggest particular sequence preferences. Furthermore, a competitive relationship exists among the frequencies of triplets that contain the same class of amino acids, which further influences frequency trends. Additionally, eigenvectors that display similar frequency trends may suggest an interaction relationship between the corresponding proteins. This indicates that there are also dependencies among these ’pseudo-sequence’ feature vectors. In the model presented in this paper, the BiGRU serves two primary functions. The first function is dimensionality reduction, the BiGRU is employed to map high-dimensional feature vectors to a more compact, lower-dimensional representation space. The second function acts as a feature converter, extracting complex and implicit sequential information from the feature vector with the assistance of the BiGRU. In this paper, we utilize the BiGRU as part of the model framework. The primary objective is to determine whether an interaction exists between two proteins. To effectively achieve this binary classification goal, we employ the binary cross-entropy loss function [42–44], which is well-suited for binary classification tasks. We supervise the training of the BiGRU using the binary classification results—indicating whether the protein pairs are interacting or not—as labels. This approach guides the hidden states in a manner that enhances the final binary classification and generates more discriminative feature vectors. In addition, to leverage the complementary advantages of deep learning models and traditional machine learning models, we subsequently input the hidden states into the LightGBM classifier rather than using the Sigmoid activation function for the final classification. Such a model design enhances the effective utilization of multi-information fusion, rather than merely generating six types of probabilistic outcomes from each of the six BiGRUs and employing a majority-voting approach for decision-making regarding the final outcome (see the S1 File Sect 5.5, S14 Table for details of the corresponding experimental results).

### Evaluation indicators

The evaluation metrics used in this study include Accuracy (ACC), Precision (PRE), Sensitivity (SE), Specificity (SP), F1 Score, and Matthew’s correlation coefficient (MCC). Additionally, ROC (Receiver Operating Characteristic) curve and PR (Precision-Recall) curve, along with the corresponding area under the curve AUC and AUPR, are also important indicators for evaluating the model performance. The formulas are detailed in the S1 File Sect 4.

## Results and discussion

### Optimization of parameter *lag*

**Fig 4:**
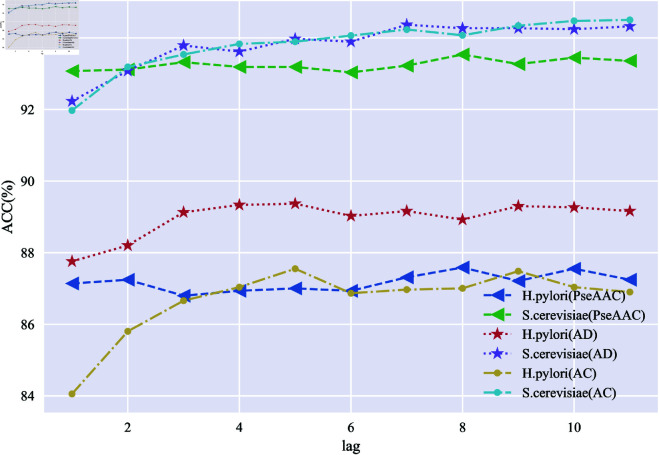
Variation in prediction accuracy of different feature coding techniques with different values of lag.

From the equations in S1 File Sect 2, it is clear that the parameters of all three feature coding techniques based on the SVHEHS descriptor need to be optimized. The shortest sequence length in the *H. pylori* dataset is 12, and the shortest sequence length in the *S. cerevisiae* dataset must be at least 50. Therefore, the *lag* can range from 1 to 11. Subsequently, the *lag* values for the three feature coding techniques, PseAAC, AD, and AC, are set accordingly. The protein pairs of the corresponding feature vectors are obtained. These feature vectors are then respectively inputted into the LightGBM classifier for PPIs prediction. The average evaluation indicator values for the five-fold cross-validation across various *lag* values are shown in S1 File Sect 5.1, S3, S4, S5, S6, S7 and S8 Tables. The model’s accuracy values under different *lag* values are illustrated in Fig 4.

From Fig 4, it can be seen that the ACC of different feature coding techniques on the two datasets are characterized by overall horizontal fluctuations at different values of the parameter *lag*. The models generally outperform the *S. cerevisiae* dataset more than the *H. pylori* dataset. The model constructed using the PseAAC feature coding technique reaches a global maximum at *lag*=8 in both the *H. pylori* and *S. cerevisiae* datasets. The model, constructed using the AD feature coding technique, performs optimally on the *H. pylori* dataset at *lag* = 5. However, the model shows optimal performance at *lag* = 7 when applied to the *S. cerevisiae* dataset for prediction. The model utilizes the AC feature coding technique to perform optimally on the *H. pylori* and *S. cerevisiae* datasets with *lag*s of 5 and 11, respectively. By comprehensively comparing the overall performance of models using various feature coding techniques across different *lag* parameter values, it is determined that the *lag* parameter values for models utilizing PseAAC, AD, and AC feature coding techniques are 8, 7, and 9, respectively. These values correspond to feature dimensions of 28, 273, and 117, respectively.

### Comparison of different feature coding techniques

**Fig 5:**
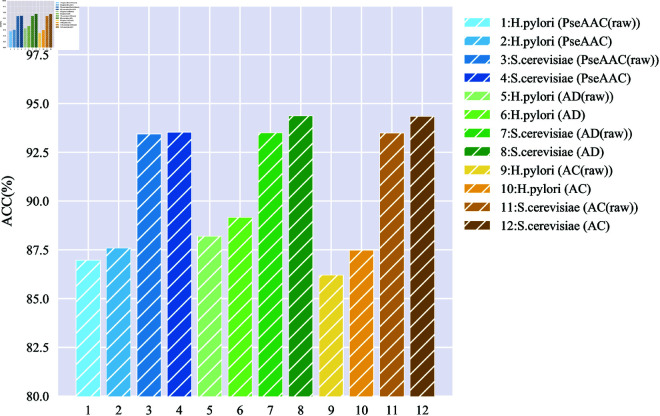
Comparison of the accuracy of different feature coding techniques.

**Fig 6:**
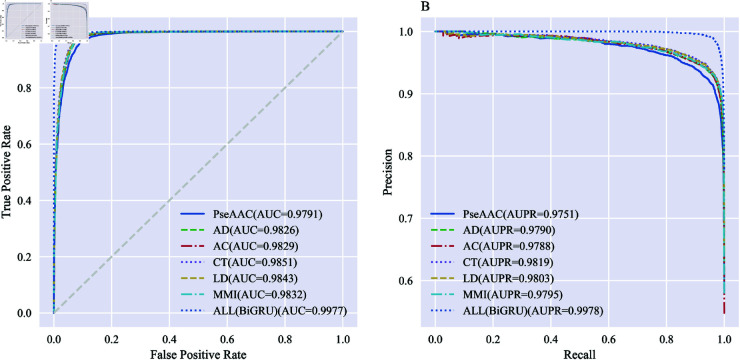
Comparison of ROC and PR curves for S. cerevisiae between multi-information fusion and single-feature information in feature coding techniques. A: ROC curves. B: PR curves.

Differences in feature coding techniques result in the final acquired feature vectors characterizing different aspects of the protein sequences, which further impact the model’s performance in predicting protein interactions. The SVHEHS descriptor is the result of a principal component analysis of the 457 physicochemical properties of 20 natural amino acids. In this paper, we present a comparison of the performance of models constructed using the SVHEHS descriptor against those utilizing feature coding techniques based on raw information on the *H. pylori* and *S. cerevisiae* datasets. The results of the five-fold cross-validation are displayed in separate tables, with detailed findings available in S1 File Sect 5.2, S9 and S10 Tables. In particular, Fig 5 presents a visual representation of how the ACC of different feature coding techniques using different descriptors varies across the two datasets. In addition, the values of the various evaluation metrics predicted by the model using the *S. cerevisiae* dataset through a multi-information fusion approach are compared to those predicted by the model based on a single feature. These results are presented in Table 1 and Fig 6, while the corresponding results for the *H. pylori* dataset are detailed in S1 File Sect 5.2, S11 Table, and S2 Fig.

**Table 1:** Prediction effectiveness of feature coding techniques with multi-information fusion and single-feature information on *S. cerevisiae.*

Feature coding techniques	ACC(%)	PRE(%)	SE(%)	SP(%)	MCC
PseAAC	93.54	95.86	91.01	96.07	0.8719
AD	94.37	96.90	91.67	97.07	0.8887
AC	94.34	97.06	91.46	97.23	0.8884
CT	94.56	97.80	91.17	97.94	0.8932
LD	94.68	97.15	92.06	97.30	0.8949
MMI	94.55	97.11	91.83	97.26	0.8923
ALL(BiGRU)	97.79	97.74	97.85	97.73	0.9559

#### Comparison of the SVHEHS descriptor-based and raw information-based.

From Fig 5 it can be seen that for different feature coding techniques, the overall performance of the model on both datasets is improved by using the SVHEHS descriptor instead of raw information. In particular, for the evaluation metric of ACC, on the *H. pylori* dataset, the model constituted by PseAAC shows a 0.62% increase in the ACC of the model constituted by PseAAC (raw), the model constituted by AD shows a 0.96% increase in the ACC of the model constituted by AD (raw), and the model constituted by AC shows a 1.27% increase in the ACC of the model constituted by AC (raw). On the *S. cerevisiae* dataset, the model constituted by PseAAC shows a 0.10% increase in the ACC of the model constituted by PseAAC(raw), the model constituted by AD shows a 0.87% increase in the ACC of the model constituted by AD (raw), and the model constituted by AC shows a 0.85% increase in the ACC of the model constituted by AC (raw).

It can be seen that the SVHEHS descriptor can characterize protein sequences more comprehensively than the raw information used in each of the original feature coding techniques. This comprehensive characterization proves to be more beneficial for models to excel in PPIs prediction. Therefore, we replace the original information with the SVHEHS descriptor and use it as part of the feature extraction by combining it with three feature coding techniques: PseAAC, AD, and AC.

#### Comparison of multi-information fusion and single-feature information.

As shown in Table 1, the prediction performance of models utilizing different feature coding techniques for PPIs prediction varies. For the *S. cerevisiae* dataset, the ACC for PseAAC, AD, AC, CT, LD, MMI, and ALL (BiGRU) are 93.54%, 94.37%, 94.34%, 94.56%, 94.68%, 94.55%, and 97.79%, respectively. When the six feature coding techniques are combined and the optimal subset of features is selected using the BiGRU, the model’s prediction effectiveness is significantly enhanced. The highest evaluated metric values of ACC, SE, and MCC are improved by 3.11%, 5.79%, and 0.0610, respectively, compared to the model consisting of a single feature.

From Fig 6, it can be seen that on the *S. cerevisiae* dataset, the model performance in order from good to bad, using AUC as an indicator, are ALL (BiGRU) (0.9977), CT (0.9851), LD (0.9843), MMI (0.9832), AC (0.9829), AD (0.9826), and PseAAC (0.9791), respectively. On the metric of AUPR, among the seven feature coding techniques (PseAAC, AD, AC, CT, LD, MMI, ALL (BiGRU)), ALL (BiGRU) has the largest values (0.9751, 0.9790, 0.9788, 0.9819, 0.9803, 0.9795 vs. 0.9978). Taken together, it can be seen that the feature coding technique with multi-information fusion outperforms the feature coding technique with single-feature information in both AUC and AUPR values. Specifically, ALL (BiGRU) demonstrates higher values compared to the other feature coding techniques by 0.0126-0.0186 and 0.0159-0.0227, respectively.

It can be seen that multi-information fusion can complement the information extracted from different feature coding techniques. This approach can characterize protein sequences more comprehensively than single-feature information and effectively improve the quality of feature vectors. Therefore, we combine six feature coding techniques: PseAAC, AD, AC, CT, LD, and MMI, to characterize the sequence and physicochemical information of protein interactions.

### Comparison of different integration methods

**Fig 7:**
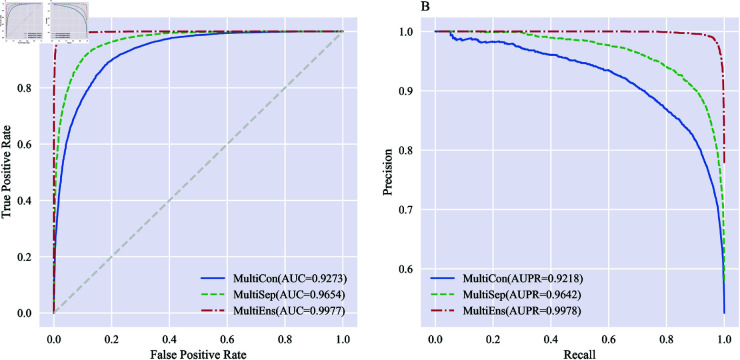
Comparison of ROC and PR curves of different integration methods on S. cerevisiae. A: ROC curves. B: PR curves.

To compare the impact of different ensemble methods of BiGRUs on the performance of a single BiGRU, this paper designs three ensemble strategies for the BiGRU. The first approach involves merging the six types of feature vectors that have undergone feature extraction, feeding them into a BiGRU with an output dimension size of 292 for the GRU layer, and inputting the optimal subset of features into LightGBM for prediction. This approach is named MultiCon (Combine multi-information in a connected way). The second approach involves dividing the feature vectors into two classes. One class is obtained using feature coding techniques based on the SVHEHS descriptor, while the other is obtained using feature coding techniques based on dipole and side chain volume. Each class of feature set corresponds to a BiGRU. That is, the feature vectors obtained by the three feature coding techniques of PseAAC, AD, and AC are concatenated and fed into a BiGRU with a GRU layer output dimension of 84. Similarly, the feature vectors obtained by the three feature coding techniques of CT, LD, and MMI are concatenated and fed into a BiGRU with a GRU layer output dimension of 208. The feature vectors of the two optimal feature subsets obtained are then merged and input into LightGBM for prediction, named MultiSep (Combine multi-information in a separate way). The third one involves associating each of the six types of feature vectors with a BiGRU. The output dimensions of the GRU layer are as follows: 4 for PseAAC, 64 for AD, 16 for AC, 64 for CT, 128 for LD, and 16 for MMI. Subsequently, the feature vectors from the six optimal feature subsets are combined and fed into LightGBM for prediction, referred to as MultiEns (Combine multi-information in an ensemble way). The prediction results from the five-fold cross-validation of the three BiGRU integration strategies for the *S. cerevisiae* dataset are compiled in Table 2 and illustrated in Fig 7. The corresponding results for the *H. pylori* dataset are presented in S1 File Sect 5.3, S12 Table, and S3 Fig.

**Table 2:** Prediction effectiveness of different integration methods on *S. cerevisiae.*

Integration method	ACC(%)	PRE(%)	SE(%)	SP(%)	MCC
MultiCon	85.06	87.40	81.93	88.18	0.7025
MultiSep	90.24	91.01	89.31	91.17	0.8050
MultiEns	97.79	97.74	97.85	97.73	0.9559

As illustrated in Table 2 and Fig 7, an increase in the number of BiGRUs across the three integration strategies resulted in an upward trend in all evaluation metrics for the *S. cerevisiae* dataset, the ACC are 85.06% for MultiCon, 90.24% for MultiSep, and 97.79% for MultiEns. In terms of AUC, the models are ranked from best to worst as MultiEns (0.9977), MultiSep (0.9654), and MultiCon (0.9273). Regarding AUPR, among the three integration strategies (MultiCon, MultiSep, MultiEns), MultiEns achieves the highest value (0.9218, 0.9642 vs. 0.9978).

It can be seen that the ensemble of independent BiGRUs improves the performance of a single BiGRU. The model tends to perform better by taking an explicit ensemble approach when controlling for a consistent size of the dimensionality of the final optimal feature subset. Therefore, we utilize the explicit ensemble of BiGRU for data dimensionality reduction to acquire the optimal feature subset.

### Comparison of different directions of GRUs

**Fig 8:**
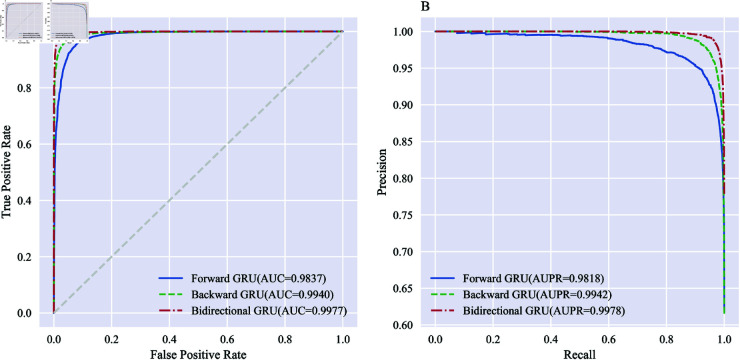
Comparison of ROC and PR curves of different directions of GRU on S. cerevisiae. A: ROC curves. B: PR curves.

The GRU can be a forward GRU or a backward GRU, depending on the direction of reading the input sequence. The bidirectional GRU is built upon this concept. In this paper, we compare the performance of three different directional GRUs in protein mutual prediction. The size of the output dimension of the GRU layer in the unidirectional GRU is set to be twice as large as that of the bidirectional GRU. This adjustment is made to ensure the dimensionality of the feature sets at the end of the three models is the same. The predicted results of the five-fold cross-validation for the *S. cerevisiae* dataset are presented in Table 3 and Fig 8. The corresponding results for the *H. pylori* dataset are detailed in S1 File Sect 5.4, S13 Table, and S4 Fig.

**Table 3:** Prediction effectiveness of GRU in different directions on *S. cerevisiae.*

Coding direction	ACC(%)	PRE(%)	SE(%)	SP(%)	MCC
Forward GRU	93.88	95.15	92.47	95.28	0.8780
Backward GRU	95.98	94.59	97.53	94.42	0.9200
Bidirectional GRU	97.79	97.74	97.85	97.73	0.9779

From Table 3 and Fig 8, it can be seen that among the three directions of the GRU, on the *S. cerevisiae* dataset, the values of the evaluation indicators are highest for the bidirectional GRU, followed by the backward GRU, with the forward GRU in third place. The ACC are 93.88% for forward GRU, 95.98% for backward GRU, and 97.79% for bidirectional GRU, respectively. In terms of the AUC metric, the model performance ranks as bidirectional GRU (0.9977), backward GRU (0.9940), and forward GRU (0.9837), in descending order of performance quality. Regarding the AUPR metric, among the three ensemble strategies (forward GRU, backward GRU, and bidirectional GRU), bidirectional GRU achieves the highest value (0.9818, 0.9942 vs. 0.9978).

It can be seen that the backward GRU outperforms the forward GRU in protein prediction, and the bidirectional GRU outperforms the unidirectional GRU when controlling for the same size of the final optimal feature subset dimension. The information encoded by forward GRU and backward GRU is complementary, and the feature vectors obtained from both of them can be combined by concatenation to characterize the protein sequence information more comprehensively. Therefore, we employ the bidirectional GRU as a tool for data dimensionality reduction.

### Comparison with traditional classifiers

To construct effective PPIs prediction models, the choice of classifier is crucial. To select the optimal classifiers, we compare Gaussian Naïve Bayes (GNB), K Nearest Neighbors (KNN), Support Vector Machines (SVM), Random Forest (RF), Logistic Regression (LR), Adaptive Boosting Algorithm (AdaBoost), Extreme Gradient Boosting (XGBoost), Extreme Random Trees (Extra-Trees), and Light Gradient Boosting Machine Learning (LightGBM). Where the parameters of LightGBM are set as described in the previous section. All classifiers, except GNB and KNN, have a random seed of 1. Additionally, the models utilize default settings. The prediction results of different classifiers on *H. pylori* and *S. cerevisiae* datasets are obtained by five-fold cross-validation, as shown in S1 File Sect 5.6, S15 and S16 Tables, S5 and S6 Figs. To assess the robustness of the classifiers further, we plot box plots illustrating the ACC of various classifiers on two datasets using five-fold cross-validation, as shown in S1 File Sect 5.6, S7 and S8 Figs. To more fully characterize the performance of the different classifiers, we also compile the running times of the different classifiers in S1 File Sect 5.6, S17 Table. A comprehensive analysis of the indicator values reveals that LightGBM exhibits superior overall performance, characterized by exceptional model accuracy, consistent prediction results, and efficient runtime. Therefore, we have ultimately selected LightGBM as the classifier for PPIs prediction.

### Comparison with different advanced models

To comprehensively evaluate the advantages and disadvantages of the model proposed in this paper, this paper compares its performance with that of state-of-the-art models proposed by other researchers on the corresponding datasets. To ensure the scientific validity of the comparison, all evaluation index values are derived from models based on five-fold cross-validation, and the data presented are sourced from the original authors’ studies. The results are compiled to create Table 4 and Table 5. More details can be found in S1 File Sect 5.7, S18, S19, S20 and S21 Tables.

**Table 4:** Prediction effectiveness of different advanced models on *H. pylori.*

Model	ACC(%)	PRE(%)	SE(%)	MCC
LPP+RF [18]	92.56±0.86	94.11±0.99	90.82±0.93	0.8622±0.0147
MatPCA+WSRC [45]	83.64±1.15	89.71±0.79	75.98±1.63	0.7226±0.0149
GTB-PPI [46]	90.47±0.84	89.99±2.06	91.15±1.42	0.8100±0.0163
Gabor+RF [47]	86.45±0.90	88.51±0.82	83.82±1.55	0.7653±0.0130
GcForest-PPI [17]	89.26±1.07	88.95±1.36	89.71±2.26	0.7857±0.0212
Our model	96.47±0.17	96.38±0.55	96.57±0.72	0.9294±0.0034

**Table 5:** Prediction effectiveness of different advanced models on *S. cerevisiae.*

Model	ACC(%)	PRE(%)	SE(%)	MCC
LPP+RF [18]	92.81±0.66	96.80±0.68	88.55±0.95	0.8661±0.0115
MatPCA+WSRC [45]	94.55±0.63	92.33±0.91	97.15±0.42	0.8968±0.0112
GTB-PPI [46]	95.15±0.25	97.97±0.60	92.21±0.36	0.9045±0.0053
Gabor+RF [47]	92.10±0.29	93.85±0.69	90.09±0.86	0.8543±0.0049
GcForest-PPI [17]	95.44±0.18	98.05±0.25	92.72±0.44	0.9102±0.0035
Our model	97.79±0.22	97.74±0.41	97.85±0.48	0.9559±0.0044

As can be seen from Table 4, on the *H. pylori* dataset, the prediction results of our model rank first among the compared methods. The results from the five-fold cross-validation show minimal variation, indicating that the model performs well and is stable.

As shown in Table 5, on the *S. cerevisiae* dataset, using PRE as the performance indicator, GcForest-PPI(98.05% ± 0.25%) achieves the highest performance. GTB-PPI(97.97% ± 0.60%) follows in second place, while our model(97.74% ± 0.41%) secures third place. However, our model excels as the top performer across all three metrics: ACC, SE, and MCC.

It can be seen that the model proposed in this paper is competitive among the existing state-of-the-art models, and the model performs well in protein interaction prediction.

### Case study

**Fig 9:**
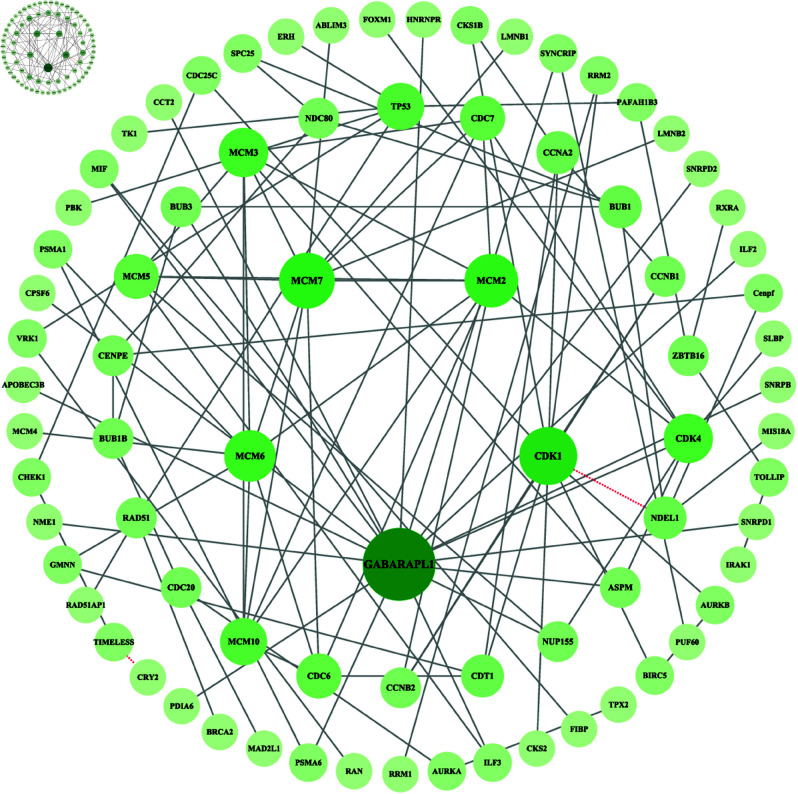
Prediction results of Disease-specific.

The robustness of model is also a major aspect of how well the model performs. The model proposed in this paper trained on the *S. cerevisiae* dataset is tested on other types of protein interaction datasets to assess the generalization performance of the model, with the results compiled in Tables 6 and 8. To visualize the performance of the model proposed in this paper on the second type of test set, a diagram of the protein-protein interaction network is drawn using the *Disease-specific* network as an example, as shown in Fig 9. In addition, the results of the models trained on the *H. pylori* dataset and evaluated on the test set are compiled alongside the corresponding results from the *S. cerevisiae* dataset in the S1 File Sect 5.8, S22 and S23 Tables. This comparison highlights the impact of training set size on model performance. The test results predicted by the trained model using the *H. pylori* dataset for both types of test sets are unsatisfactory. Our analysis indicates that the limited amount of data in the *H. pylori* dataset—less than that found in some test sets—likely hindered the model’s ability to train effectively. This limitation may have contributed to issues such as overfitting, poor generalization, and unstable training. In particular, the BiGRU is utilized for feature transformation within the overall framework. A stable training process is crucial for deep learning models, as they depend on large volumes of data to identify consistent and reliable patterns. Besides, this paper also compares the predictive performance of the model proposed in this paper on the test set with various advanced models, as shown in Table 7. All evaluation metric values represent predictions based on the corresponding test set after the model was trained on the *S. cerevisiae* dataset. The data presented are derived from the original authors’ study.

**Table 6:** Prediction effectiveness on the first type of test set.

Dataset	ACC(%)	SE(%)	F1(%)
*C. elegans*	94.14	94.14	96.98
*E. coli*	96.89	96.89	98.42
*H. sapiens*	95.96	95.96	97.94
*M. musculus*	94.89	94.89	97.38

**Table 7:** Prediction accuracy of different advanced models on the first type of test set.

Model	*C. elegans*	*E. coli*	*H. sapiens*	*M. musculus*
DeepPPI [48]	94.84	92.19	93.77	91.37
LightGBM-PPI [37]	90.16	92.16	94.83	94.57
MatPCA+WSRC [45]	96.84	90.14	97.10	95.21
GTB-PPI [46]	92.42	94.06	97.38	98.08
EnsDNN [49]	93.22	95.10	95.00	94.06
Our model	94.14	96.89	95.96	94.89

**Table 8:** Prediction effectiveness on the second type of test set.

Dataset	ACC(%)	SE(%)	F1(%)
*Disease-specific*	98.15	98.15	99.07
*One-core network*	100.00	100.00	100.00
*Wnt-related pathway*	100.00	100.00	100.00

#### Protein interaction network prediction for the first type of test set.

The first type of test set consists of four cross-species datasets, namely *C. elegans*, *E. coli*, *H. sapiens*, and *M. musculus*.

As can be seen from Table 6, our model still maintains good prediction performance on the four cross-species datasets. The ACC on the *C. elegans*, *E. coli*, *H. sapiens*, and *M. musculus* datasets are 94.14%, 96.89%, 95.96%, and 94.89%, respectively. The F1 scores are 96.98%, 98.42%, 97.94%, and 97.38%, respectively.

From Table 7, it can be seen that our model has the highest ACC on the *E. coli* dataset at 96.89% compared to various state-of-the-art models on four independent test sets. On the *C. elegans* dataset, MatPCA+WSRC achieves the highest accuracy of 96.84%, with our model at 94.14% in third place. On both the *H. sapiens* and *M. musculus* datasets, the highest ACC are found for GTB-PPI (97.38% and 98.08%), with our model at 95.96% and 94.89%, respectively, both in third place.

It can be seen that the model proposed in this paper, compared to existing advanced models, is not the best predictor on the four independent test sets. However, the ACC can still be maintained at a high level, indicating the robustness of the model.

#### Protein interaction network prediction for the second type of test set.

The second type of test set includes *Disease-specific*, *One-core network*, and *Wnt-related pathway*. *Disease-specific* is composed of 78 genes. *One-core network* is a simple network of mononuclear protein interactions consisting of 17 genes and with CD9 as the core protein. CD9 is a tetraspanin that plays a crucial role in epidermal growth factor receptor signaling and tumor suppression [31]. *Wnt-related pathway* contains 78 genes. Wnt is a secreted glycoprotein that plays an important role in embryogenesis and cortical development.

As can be seen from Table 8 and Fig 9, our model still maintains good prediction performance on the disease-related PPIs dataset. In the *Disease-specific* category, our model successfully predicts 106 out of 108 pairs of protein interactions with 98.15% accuracy. It also accurately predicts 16 pairs of protein interactions in the mononuclear network and 96 pairs of protein interactions in the *One-core network* and *Wnt-related pathway*. Of course, the exceptionally high prediction accuracy is closely related to the limited amount of data in the dataset.

The model proposed in this paper exhibits strong generalization capabilities, robust performance, and the potential to inspire innovative research ideas.

## Conclusion

In this paper, the model proposed in this paper is identified through filtering. This is achieved by selecting the strategy with the best prediction effect in each module through side-by-side comparison at different stages. Our model involves inputting the feature vectors obtained from various feature coding techniques into different BiGRUs for separate data dimensionality reduction. The output dimensions of each GRU layer are determined based on the dimensionality of the feature vectors obtained from each feature coding technique. Subsequently, the merged optimal feature set is fed into LightGBM to predict protein interactions.

Several aspects of the proposed approach are worth highlighting. (1) The SVHEHS descriptor allows for a more comprehensive characterization of information about a protein sequence than the raw information used by each of the three feature coding techniques: PseAAC, AD, and AC. (2) The multi-information fusion approach effectively complements the information extracted by a single-feature information coding technique and can significantly enhance the quality of the feature vector. (3) The output dimension of the GRU layer in the BiGRU is determined based on the dimension of the input feature vectors and a specific computational rule. (4) The backward GRU outperforms the forward GRU, and the bidirectional GRU outperforms the unidirectional GRU when the dimensional size of the optimal feature set after dimensionality reduction is the same. (5) When the dimensional size of the optimal feature set after dimensionality reduction is the same, integrating independent BiGRUs can enhance the performance of a single BiGRU. This implies that the model performs better when an explicit ensemble approach is adopted. The final model performs well on the training set, with stable and accurate model predictions, short running time, and high computational efficiency. It also demonstrates effectiveness on the test set and exhibits strong generalization ability, indicating its potential to provide new ideas and insights for exploring protein interaction networks and disease-related genes.

Although our experimental results demonstrate that the model proposed in this paper exhibits greater stability and performance, several aspects require improvement in the future: (1) This paper focuses exclusively on protein sequence information; incorporating structural data could enhance the quality of the feature set. In particular, pre-trained protein language models [23–25] show significant potential for advancing feature extraction. (2) The current approach utilizes a straightforward serial linkage to integrate feature information. The BiGRU could be enhanced by incorporating optimization algorithms, attention mechanisms, regularization techniques, and custom loss functions [15,22]. (3) The interpretability of the dataset following dimensionality reduction of the BiGRU presents a significant challenge, which could be addressed through post-processing methods such as feature importance analysis.

## Supporting information

S1 File All supporting information are collated in this word document (DOCX)
