# Supplementary material for: Protein-protein interaction prediction using bidirectional GRUs with explicit ensemble
Source: PLoS One. 2025 Jul 2;20(7):e0326960. doi: 10.1371/journal.pone.0326960 (PMC12221175; doi:10.1371/journal.pone.0326960)
Supplement: S1 File — (DOCX) [file pone.0326960.s001.docx]

**Supporting information**

**Catalogue**

[1. The SVHEHS descriptor for 20 natural amino acids 2](#_Toc199950166)

[2. Feature coding technique formula 2](#_Toc199950167)

[2.1 Based on the SVHEHS descriptor 2](#_Toc199950168)

[2.1.1 Pseudo Amino Acid Composition (PseAAC) 2](#_Toc199950169)

[2.1.2 Autocorrelation Descriptor (AD) 4](#_Toc199950174)

[2.1.3 Auto-Covariance (AC) 4](#_Toc199950178)

[2.2 Based on dipole and side chain volume 4](#_Toc199950180)

[2.2.1 Conjoint Triad (CT) 5](#_Toc199950181)

[2.2.2 Local Descriptor (LD) 5](#_Toc199950183)

[2.2.3 Multivariate Mutual Information (MMI) 5](#_Toc199950184)

[3. Model description 6](#_Toc199950192)

[3.1 Bidirectional GRU 6](#_Toc199950193)

[3.2 LightGBM 8](#_Toc199950198)

[4. Evaluation indicators 9](#_Toc199950199)

[5. Supplementary results 10](#_Toc199950206)

[5.1 Optimization of parameter *lag* 10](#_Toc199950207)

[5.2 Comparison of different feature coding techniques 12](#_Toc199950208)

[5.3 Comparison of different integration methods 13](#_Toc199950209)

[5.4 Comparison of different directions of GRUs 14](#_Toc199950210)

[5.5 Classification Results Using the BiGRU 14](#_Toc199950211)

[5.6 Comparison with traditional classifiers 14](#_Toc199950212)

[5.7 Comparison with different advanced models 17](#_Toc199950213)

[5.8 Comparison of test results across two training datasets 18](#_Toc199950214)

# The SVHEHS descriptor for 20 natural amino acids

S Table. The SVHEHS descriptor for 20 natural amino acids.

| Code | Score Vector SVHEHS | | | | | | | | | | | | |
| --- | --- | --- | --- | --- | --- | --- | --- | --- | --- | --- | --- | --- | --- |
|  | hydrophobic  properties | | electronic  properties | | | | hydrogen bonds  contributions | | steric  properties | | | | |
|  | 1 | 2 | 3 | 4 | 5 | 6 | 7 | 8 | 9 | 10 | 11 | 12 | 13 |
| A | 0.65 | -5.37 | -1.61 | -1.82 | -0.46 | 0.44 | -1.89 | 0 | 2.53 | 9.12 | 9.91 | -0.8 | -4 |
| C | 7.08 | -4.93 | 0.47 | 2.48 | 3.63 | -0.59 | -1.65 | 1.54 | 2.03 | -9.24 | -3.27 | -5.5 | -10.81 |
| D | -9.56 | -0.88 | 6.78 | -2.49 | 2.45 | 0.26 | 1.21 | -0.15 | -12.37 | 5.21 | -2.71 | -1.19 | -2.07 |
| E | -9.61 | 0.43 | 3.77 | -4.56 | -0.69 | 0.64 | 1.38 | 1.24 | -2.57 | 16.03 | -0.98 | 1.27 | -3.87 |
| F | 12.35 | 2.81 | -0.94 | 2.4 | 2.35 | 0.43 | -1.36 | -0.27 | 11.09 | -4.08 | -0.9 | 0.38 | 0.7 |
| G | -2.46 | -7.21 | -1.4 | -4 | -2.17 | 0.37 | -2.1 | -0.74 | -18.02 | -6.73 | 10 | -7.69 | 2.82 |
| H | -3.21 | 1.47 | 1.67 | 2.42 | -0.99 | -1.41 | 0.42 | 0.14 | 2.14 | 0.29 | -7.66 | -3.62 | -0.64 |
| I | 12.46 | -0.57 | -3.79 | -1.44 | -0.52 | 0.62 | -1.08 | -0.73 | 13.53 | -5.41 | 5.26 | 4.05 | 1.36 |
| K | -10.97 | 3.46 | 0.56 | 2.01 | -4.51 | 0.36 | 2.13 | -1.62 | -2.73 | 10.16 | -2.99 | 0.33 | 6.87 |
| L | 11.27 | -0.36 | -3.3 | -1.27 | -0.26 | 0.9 | -1.01 | -1.75 | 12.1 | 4.57 | 10.13 | 1.3 | 1.18 |
| M | 7.49 | -0.88 | -1.24 | 1.05 | 1.46 | 1.08 | -0.97 | -1.06 | 13.35 | 3.09 | -3.33 | -1.81 | -4.09 |
| N | -8.26 | -0.55 | 2.36 | -0.28 | 0.15 | 0.03 | 1.69 | -0.19 | -11.37 | -0.99 | -2.85 | -5.03 | 1.89 |
| P | -1.97 | -1.3 | -1.76 | -1.08 | 0.39 | -6.4 | -1.62 | 1.75 | -18.4 | -5.85 | -2.14 | 14.8 | -4.45 |
| Q | -8.41 | 1.03 | 1.05 | 0.32 | -0.44 | 1 | 1.88 | 0.82 | -0.95 | 5.3 | -4.69 | 0.99 | -0.35 |
| R | -11.92 | 6.82 | 2.82 | 4.48 | -4.08 | -0.22 | 4.29 | 0.05 | -0.35 | 3.88 | -5.45 | 1.8 | 6.41 |
| S | -5.42 | -3.62 | 0.73 | -0.49 | 0.98 | -0.21 | -0.12 | 0.68 | -10.47 | -2.03 | 4.7 | -1.8 | 0.95 |
| T | -3.12 | -2.47 | -0.05 | 0.02 | 0.26 | -0.07 | 0.17 | 0.07 | -3.37 | -4.17 | 3.58 | 0.19 | 0.68 |
| V | 8.88 | -2.71 | -3.26 | -1.6 | -0.62 | 0.54 | -1.4 | 0.3 | 11.34 | -5.1 | 9.27 | 2.86 | 0.93 |
| W | 10.99 | 8.5 | -1.93 | 2.28 | 1.83 | 1.51 | -0.22 | -1.38 | 9.84 | -4.52 | -10.5 | -1.44 | -0.09 |
| Y | 3.73 | 6.32 | -0.92 | 1.56 | 1.25 | 0.72 | 0.24 | 1.3 | 2.65 | -9.55 | -5.39 | 0.93 | 6.59 |

# Feature coding technique formula

## Based on the SVHEHS descriptor

### Pseudo Amino Acid Composition (PseAAC)

The feature vector of the PseAAC feature coding technique can be represented as

| $X=[x_{1},x_{2},x_{3},\cdots,x_{20},x_{21},\cdots,x_{20+lag}],lag<L,$ |  |
| --- | --- |

where the first 20-dimensional feature vector represents the compositional information of amino acids, the latter $lag$-dimensional feature vector represents the sequential information of amino acids. $L$ is the length of the protein sequence, while $lag$ is a parameter that is yet to be determined.

$x_{k}(k=1,2,\cdots,20+lag)$ is defined as follows

| $x_{k}=\left\{\begin{matrix} \frac{f_{k}}{\sum_{i=1}^{20} f_{i}+\omega\sum_{j=1}^{lag} \theta_{j}},1\leq k\leq20, \\ \frac{\omega\theta_{k-20}}{\sum_{i=1}^{20} f_{i}+\omega\sum_{j=1}^{lag} \theta_{j}},21\leq k\leq20+lag, \end{matrix}\right.$ |  |
| --- | --- |

where $f_{i}(i=1,2,\cdots,20)$ represents the frequency of occurrence of the 20 natural amino acids in the sequence, $\omega=0.05$ represents the weight factor, $\theta_{j}(j=1,2,\cdots,lag)$ represents the $j-th$ order sequence correlation factor, and $lag$ is a parameter to be determined.

$\theta_{j}(j=1,2,\cdots,lag)$ is defined as follows

| $\left\{\begin{matrix} \theta_{1}=\frac{1}{L-1}\sum_{i=1}^{L-1} \Theta(i,i+1), \\ \theta_{2}=\frac{1}{L-2}\sum_{i=1}^{L-2} \Theta(i,i+2), \\ \theta_{3}=\frac{1}{L-3}\sum_{i=1}^{L-3} \Theta(i,i+3), \\ \cdots\cdots\\ \theta_{lag}=\frac{1}{L-lag}\sum_{i=1}^{L-lag} \Theta(i,i+lag), \end{matrix}\right.lag<L,$ |  |
| --- | --- |

where $\Theta(i,j)$ is defined as follows

| $\Theta(i,j)=\frac{1}{13}\left\{[H_{1}(j)-H_{1}(i)]^{2}+[H_{2}(j)-H_{2}(i)]^{2}+\cdots+[H_{13}(j)-H_{13}(i)]^{2}\right\}$ |  |
| --- | --- |

where $H_{i}(j)(i=1,2,\cdots,13;j=1,2,\cdots,L)$ denotes the $i-th$ SVHEHS descriptor value of the $j-th$ amino acid.

### Autocorrelation Descriptor (AD)

| $MBroto(l)=\frac{\sum_{i=1}^{L-l} A_{i,j}A_{(i+l),j}}{L-l},l=1,2,\cdots,lag;j=1,2,\cdots,13,$ |  |
| --- | --- |

| $Moran(l)=\frac{\frac{1}{L-l}\sum_{i=1}^{L-l} (A_{i,j}-\overline{A_{j}})(A_{(i+l),j}-\overline{A_{j}})}{\frac{1}{L}\sum_{i=1}^{L} (A_{i,j}-\overline{A_{j}})^{2}},l=1,2,\cdots,lag;j=1,2,\cdots,13,$ |  |
| --- | --- |

| $Geary(l)=\frac{\frac{1}{2(L-l)}\sum_{i=1}^{L-l} (A_{i,j}-A_{(i+l),j})^{2}}{\frac{1}{L}\sum_{i=1}^{L} (A_{i,j}-\overline{A_{j}})^{2}},l=1,2,\cdots,lag;j=1,2,\cdots,13,$ |  |
| --- | --- |

where $L$ is the length of the protein sequence, $lag$ is the parameter to be determined; $A_{i,j}$ denotes the value of the $j-th$ SVHEHS descriptor for the $i-th$ amino acid; and $\overline{A_{j}}$ denotes the average value of the $j-th$ SVHEHS descriptor.

### Auto-Covariance (AC)

| $AC_{l,j}=\frac{\sum_{i=1}^{L-l} (A_{i,j}-\frac{\sum_{i=1}^{L} A_{i,j}}{L})(A_{(i+l),j}-\frac{\sum_{i=1}^{L} A_{i,j}}{L})}{L-l},l=1,2,\cdots,lag;j=1,2,\cdots,13,$ |  |
| --- | --- |

where $L$ represents the length of the protein sequence, $lag$ is the parameter to be determined, and $A_{i,j}$ denotes the $j-th$ SVHEHS descriptor value for the $i-th$ amino acid.

## Based on dipole and side chain volume

S Table. Amino acid classification based on dipole and side chain volume.

| Group | Amino Acid | Dipoles | Volumes of Side Chains |
| --- | --- | --- | --- |
| 1 | A, G, V | Dipole<1.0 | volume<50 |
| 2 | C | 1.0<dipole<2.0(form disulphide bonds) | volume>50 |
| 3 | D, E | dipole>3.0(opposite orientation) | volume>50 |
| 4 | F, I, L, P | dipole<1.0 | volume>50 |
| 5 | H, N, Q, W | 2.0<dipole<3.0 | volume>50 |
| 6 | K, R | Dipole>3.0 | volume>50 |
| 7 | M, S, T, Y | 1.0<dipole<2.0 | volume>50 |

### Conjoint Triad (CT)

| $CT_{i}=\frac{g_{i}-\min\{g_{1},g_{2},\cdots,g_{343}\}}{\max\{g_{1},g_{2},\cdots,g_{343}\}},i=1,2,\cdots,343,$ |  |
| --- | --- |

where $g_{i}(i=1,2,\cdots,343)$ represents the frequency of occurrence of the $i-th$ triplex in the protein sequence.

### Local Descriptor (LD)

Composition (C): Frequency of occurrence of each type of amino acid in the subsequence.

Transition (T): The frequency with which a dipeptide, composed of amino acids from two different classes, occurs in a subsequence.

Distribution (D): The proportion of sequence lengths of each type of amino acid at positions 1, 25%, 50%, 75%, and 100% in the subsequence.

### Multivariate Mutual Information (MMI)

The mutual information definition of the 3-gram feature is

| $I(a,b,c)=I(a,b)-I(a,b\vert c),a,b,c=1,2,\cdots,7,$ |  |
| --- | --- |

where $a,b,c$ represent three consecutive amino acids in the protein sequence, $I(a,b)$ denotes the mutual information of the 2-gram feature, and $I(a,b|c)$ represents the conditional mutual information.

The definition of mutual information for 2-gram features is

| $I(a,b)=f(a,b)\ln[\frac{f(a,b)}{f(a)f(b)}],a,b=1,2,\cdots,7,$ |  |
| --- | --- |

where $a,b$ represent two consecutive amino acids in the protein sequence, $f(a),f(b)$ denote the frequency of class $a$ and class $b$ amino acids in the protein sequence, respectively, and $f(a,b)$ denotes the frequency of class $a$ and class $b$ amino acids in the 2-gram feature.

Conditional mutual information is defined as

| $I(a,b\vert c)=H(a\vert c)-H(a\vert b,c),a,b,c=1,2,\cdots,7,$ |  |
| --- | --- |

where $H(a|c),H(a|b,c)$ represent the conditional entropy.

$H(a|c)$ is defined as follows

| $H(a\vert c)=-f(a\vert c)\ln[f(a\vert c)],a,c=1,2,\cdots,7,$ |  |
| --- | --- |

where $f(a|c)$ represents the frequency of occurrence of amino acids of class $a$ based on the presence of amino acids of class $c$ in the 2-gram feature, and $H(a|c)$ can be approximated as

| $H(a\vert c)=-\frac{f(a,c)}{f(c)}\ln[\frac{f(a,c)}{f(c)}],a,c=1,2,\cdots,7,$ |  |
| --- | --- |

$H(a|b,c)$ is defined as follows

| $H(a\vert b,c)=-f(a\vert b,c)\ln[f(a\vert b,c)],a,b,c=1,2,\cdots,7,$ |  |
| --- | --- |

where $f(a|b,c)$ denotes the frequency of occurrence of amino acids of class $a$ based on the presence of amino acids of class $b$ and $c$ in the 3-gram characterization, and $H(a|b,c)$ can be approximated as

| $H(a\vert b,c)=-\frac{f(a,b,c)}{f(b,c)}\ln[\frac{f(a,b,c)}{f(b,c)}],a,b,c=1,2,\cdots,7,$ |  |
| --- | --- |

where $f(a,b,c)$ denotes the frequency of occurrence of class $a$, $b$, and $c$ amino acids in the 3-gram feature.

# Model description

## Bidirectional GRU


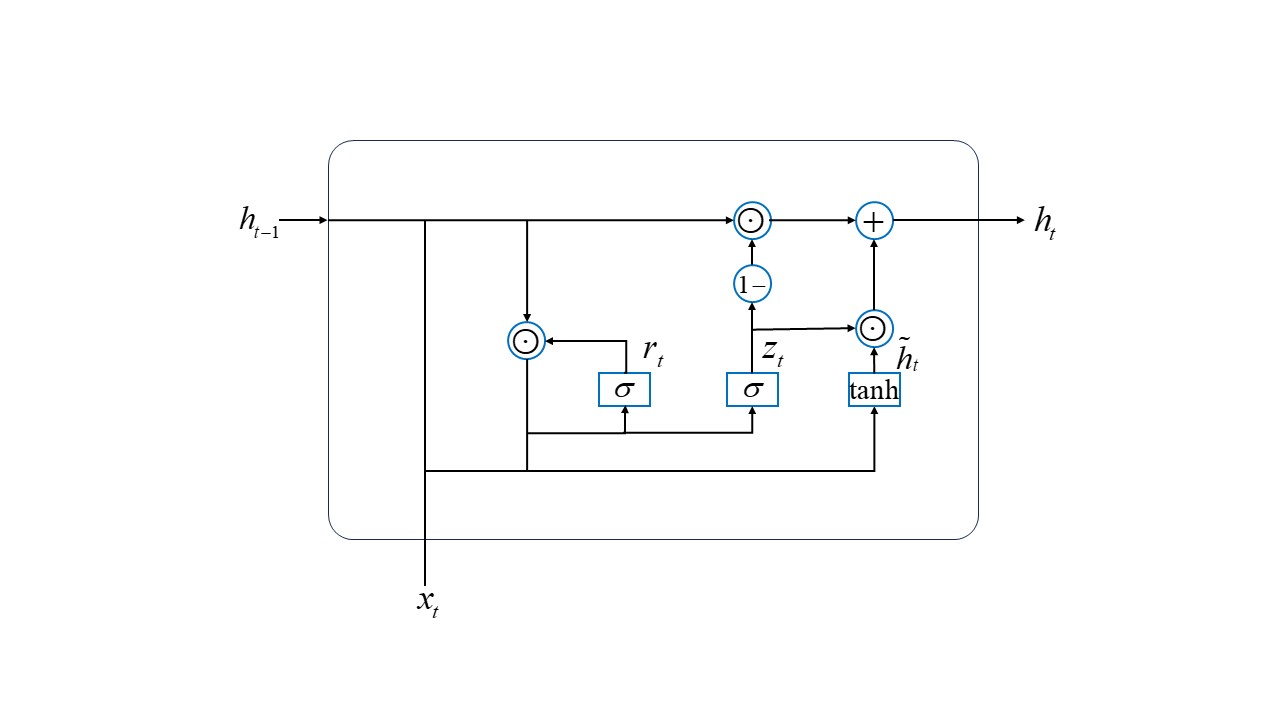


S Fig. Gated recurrent unit model

The gated recurrent unit (GRU) model [1] is shown in S1 Fig. It is a successful variant of the long short-term memory (LSTM) neural network. GRU makes two major changes to LSTM. One change is to transform the input, forget, and output gates into two gates: update and reset gates. The other change is to combine the cell state and output into one state. The two major changes simplify the network structure and reduce the number of parameters. This allows the recurrent neural network (RNN) to handle long-term dependencies effectively while also lowering operating costs and enhancing efficiency. These advantages are particularly evident in processing long sequential data. GRU is extensively utilized in natural language processing, speech recognition, machine translation, and other domains.

GRU updates the hidden state $h$ of the sequence by computing a linear combination of the previous state $h_{t-1}$ and the candidate state $\widetilde{h}_{t}$. The detailed formula is as follows.

| $h=(1-z_{t})\odot h_{t-1}+z_{t}\odot\widetilde{h_{t}},$ |  |
| --- | --- |

| $z_{t}=\sigma(W_{z}\cdot[h_{t-1},x_{t}]),$ |  |
| --- | --- |

| $\widetilde{h}_{t}=\tanh(W\cdot[r_{t}\odot h_{t-1},x_{t}]),$ |  |
| --- | --- |

| $r_{t}=\sigma(W_{r}\cdot[h_{t-1},x_{t}]),$ |  |
| --- | --- |

Where $z_{t}$ represents the update gate, balancing the previous state $h_{t-1}$ and the current input $x_{t}$; $r_{t}$ represents the reset gate, controlling the contribution of the previous state $h_{t-1}$ to the candidate state $\widetilde{h}_{t}$. $W_{*}$ and $b_{*}$ represent the weight matrix and the bias vector, respectively. The symbol $\sigma$ denotes the sigmoid function, and represents the element-by-element multiplication.

Whereas BiGRU (Bidirectional GRU) characterizes sequence information in two directions [2]. In BiGRU, the input sequence is passed to two separate GRU models simultaneously. One model is responsible for forward coding $\overrightarrow{GRU}$, while the other handles backward coding $\overleftarrow{GRU}$. The GRU model for each direction learns to capture contextual information in different directions and then combines the information from both directions to provide a more comprehensive representation. In other words, the encoded results from both directions are concatenated to each input term $x_{t}$, ultimately yielding $h=BiGRU_{(x_{t})}=[\overrightarrow{GRU}_{(x_{t})},\overleftarrow{GRU}_{(x_{t})}]$.

## LightGBM

LightGBM [3] is an improved algorithm for the defects of XGBoost [4], which solves the problem of high model complexity due to the number of samples, features, and splitting points when finding the optimal feature splitting point in XGBoost, so it is also an engineering implementation of the GBDT algorithm.

Distinguishing from the pre-ranking algorithm of XGBoost, LightGBM employs a histogram algorithm to find the optimal feature splitting point by storing continuous feature values into discrete bins and using these bins to construct the histogram during training. Thus the computational cost of finding feature segmentation points drops from O(#data × #feature) to O(#bin × #feature), where #bin<<#data.

To reduce the number of samples, LightGBM designs the one-sided gradient sampling algorithm (GOSS) to eliminate a large percentage of small gradient data instances and use only the remaining samples to estimate the information gain, where small gradient data instances are samples with small weights. The samples are first sorted according to the absolute value of the sample gradient, followed by selecting the top a × 100% of the samples and randomly sampling b × 100% of the samples from the remaining data. Finally, when calculating the information gain, the small gradient data instances are scaled up by a constant ($\frac{1-a}{b}$) to focus more attention on the under-trained samples without changing too much of the original data distribution.

High-dimensional data tends to be sparse, and in a sparse feature space, many features are mutually exclusive, meaning that they do not take on non-zero values simultaneously. LightGBM introduces the mutually exclusive feature bundle (EFB) algorithm to reduce the dimensionality of features. Mutually exclusive features are bundled into a single feature, and a feature scanning algorithm is used to construct feature histograms from the feature package that are identical to the feature histograms from the individual features.

In addition, LightGBM discards XGBoost's level-wise algorithm of growing by layers and uses a depth-constrained algorithm of growing by leaf nodes (leaf-wise). The strategy finds the leaf with the largest splitting gain from all the current leaves to split each time, and the cycle continues. In contrast, the leaf-wise algorithm can save computational costs, and together with the depth limiting mechanism, it can effectively prevent model overfitting while ensuring computational accuracy.

LightGBM has made some optimizations in its engineering implementation, such as direct support for category features, cache hit rate optimization, and so on. In general, compared with XGBoost, LightGBM is an efficient, lightweight, and scalable framework for gradient boosting trees with faster training speed and lower memory consumption while ensuring that accuracy is not degraded.

# Evaluation indicators

| $ACC=\frac{TP+TN}{TP+TN+FP+FN},$ |  |
| --- | --- |

| $PRE=\frac{TP}{TP+FP},$ |  |
| --- | --- |

| $SE=\frac{TP}{TP+FN},$ |  |
| --- | --- |

| $SP=\frac{TN}{TN+FP},$ |  |
| --- | --- |

| $F1=\frac{2\times PRE\times SE}{PRE+SE}=\frac{2TP}{2TP+FP+FN},$ |  |
| --- | --- |

| $MCC=\frac{(TP\times TN-FP\times FN)}{\sqrt{(TP+FP)\times(TP+FN)\times(TN+FP)\times(TN+FN)}},$ |  |
| --- | --- |

where TP, TN, FP, and FN are true positive, true negative, false positive, and false negative, respectively.

# Supplementary results

## Optimization of parameter *lag*

S Table. Predictive effectiveness of the PseAAC feature coding technique on *H. pylori* at different *lag* values.

| lag | ACC(%) | PRE(%) | SE(%) | SP(%) | MCC |
| --- | --- | --- | --- | --- | --- |
| 1 | 87.14 | 87.32 | 86.90 | 87.38 | 0.7430 |
| 2 | 87.24 | 86.95 | 87.65 | 86.83 | 0.7451 |
| 3 | 86.80 | 86.93 | 86.63 | 86.97 | 0.7361 |
| 4 | 86.93 | 87.47 | 86.21 | 87.65 | 0.7390 |
| 5 | 87.00 | 86.86 | 87.17 | 86.83 | 0.7403 |
| 6 | 86.93 | 87.07 | 86.76 | 87.11 | 0.7389 |
| 7 | 87.31 | 87.37 | 87.24 | 87.38 | 0.7463 |
| 8 | 87.59 | 87.54 | 87.65 | 87.52 | 0.7520 |
| 9 | 87.21 | 87.39 | 86.97 | 87.45 | 0.7442 |
| 10 | 87.55 | 87.49 | 87.65 | 87.45 | 0.7513 |
| 11 | 87.24 | 87.20 | 87.31 | 87.17 | 0.7452 |

S Table. Predictive effectiveness of the PseAAC feature coding technique on *S. cerevisiae* at different *lag* values.

| lag | ACC(%) | PRE(%) | SE (%) | SP(%) | MCC |
| --- | --- | --- | --- | --- | --- |
| 1 | 93.07 | 95.21 | 90.70 | 95.44 | 0.8624 |
| 2 | 93.12 | 95.42 | 90.58 | 95.66 | 0.8635 |
| 3 | 93.32 | 95.67 | 90.76 | 95.89 | 0.8677 |
| 4 | 93.19 | 95.54 | 90.61 | 95.76 | 0.8650 |
| 5 | 93.19 | 95.52 | 90.63 | 95.75 | 0.8650 |
| 6 | 93.04 | 95.47 | 90.36 | 95.71 | 0.8620 |
| 7 | 93.22 | 95.49 | 90.74 | 95.71 | 0.8656 |
| 8 | 93.54 | 95.86 | 91.01 | 96.07 | 0.8719 |
| 9 | 93.27 | 95.51 | 90.81 | 95.73 | 0.8665 |
| 10 | 93.45 | 96.17 | 90.51 | 96.39 | 0.8705 |
| 11 | 93.36 | 95.93 | 90.56 | 96.16 | 0.8686 |

S Table. Predictive effectiveness of the AD feature coding technique on *H. pylori* at different *lag* values.

| lag | ACC(%) | PRE(%) | SE(%) | SP(%) | MCC |
| --- | --- | --- | --- | --- | --- |
| 1 | 87.76 | 87.81 | 87.72 | 87.79 | 0.7555 |
| 2 | 88.20 | 88.10 | 88.41 | 88.00 | 0.7646 |
| 3 | 89.13 | 88.98 | 89.37 | 88.89 | 0.7829 |
| 4 | 89.33 | 89.46 | 89.23 | 89.44 | 0.7870 |
| 5 | 89.37 | 89.76 | 88.89 | 89.85 | 0.7875 |
| 6 | 89.03 | 89.44 | 88.55 | 89.51 | 0.7807 |
| 7 | 89.16 | 89.46 | 88.82 | 89.51 | 0.7834 |
| 8 | 88.92 | 88.99 | 88.89 | 88.96 | 0.7787 |
| 9 | 89.30 | 89.61 | 88.96 | 89.64 | 0.7862 |
| 10 | 89.27 | 89.70 | 88.75 | 89.78 | 0.7855 |
| 11 | 89.16 | 89.35 | 89.03 | 89.30 | 0.7838 |

S Table. Predictive effectiveness of the AD feature coding technique on *S. cerevisiae* at different *lag* values.

| lag | ACC(%) | PRE(%) | SE(%) | SP(%) | MCC |
| --- | --- | --- | --- | --- | --- |
| 1 | 92.23 | 94.89 | 89.27 | 95.19 | 0.8462 |
| 2 | 93.08 | 96.03 | 89.88 | 96.28 | 0.8634 |
| 3 | 93.79 | 96.54 | 90.83 | 96.75 | 0.8773 |
| 4 | 93.62 | 96.37 | 90.65 | 96.59 | 0.8739 |
| 5 | 93.98 | 96.49 | 91.28 | 96.67 | 0.8808 |
| 6 | 93.90 | 96.58 | 91.01 | 96.78 | 0.8794 |
| 7 | 94.37 | 96.90 | 91.67 | 97.07 | 0.8887 |
| 8 | 94.27 | 96.77 | 91.60 | 96.94 | 0.8867 |
| 9 | 94.27 | 96.86 | 91.51 | 97.03 | 0.8868 |
| 10 | 94.24 | 96.93 | 91.38 | 97.10 | 0.8863 |
| 11 | 94.32 | 97.02 | 91.45 | 97.19 | 0.8880 |

S Table. Predictive effectiveness of the AC feature coding technique on *H. pylori* at different *lag* values.

| lag | ACC(%) | PRE(%) | SE(%) | SP(%) | MCC |
| --- | --- | --- | --- | --- | --- |
| 1 | 84.05 | 83.85 | 84.36 | 83.75 | 0.6813 |
| 2 | 85.80 | 85.25 | 86.63 | 84.98 | 0.7162 |
| 3 | 86.66 | 86.55 | 86.83 | 86.49 | 0.7333 |
| 4 | 87.04 | 86.93 | 87.24 | 86.83 | 0.7411 |
| 5 | 87.55 | 87.49 | 87.72 | 87.38 | 0.7515 |
| 6 | 86.87 | 86.80 | 87.04 | 86.70 | 0.7377 |
| 7 | 86.97 | 86.95 | 87.04 | 86.90 | 0.7397 |
| 8 | 87.00 | 87.12 | 86.90 | 87.11 | 0.7403 |
| 9 | 87.48 | 88.28 | 86.49 | 88.48 | 0.7501 |
| 10 | 87.04 | 86.81 | 87.38 | 86.69 | 0.7409 |
| 11 | 86.90 | 87.29 | 86.42 | 87.38 | 0.7382 |

S Table. Predictive effectiveness of the AC feature coding technique on *S. cerevisiae* at different *lag* values.

| lag | ACC(%) | PRE(%) | SE(%) | SP(%) | MCC |
| --- | --- | --- | --- | --- | --- |
| 1 | 91.97 | 95.10 | 88.51 | 95.44 | 0.8415 |
| 2 | 93.19 | 96.27 | 89.86 | 96.51 | 0.8657 |
| 3 | 93.54 | 96.19 | 90.67 | 96.41 | 0.8722 |
| 4 | 93.83 | 96.48 | 90.99 | 96.67 | 0.8781 |
| 5 | 93.90 | 96.80 | 90.79 | 97.00 | 0.8796 |
| 6 | 94.07 | 96.72 | 91.22 | 96.91 | 0.8827 |
| 7 | 94.23 | 96.79 | 91.51 | 96.96 | 0.8860 |
| 8 | 94.07 | 96.80 | 91.17 | 96.98 | 0.8830 |
| 9 | 94.34 | 97.06 | 91.46 | 97.23 | 0.8884 |
| 10 | 94.48 | 97.07 | 91.72 | 97.23 | 0.8909 |
| 11 | 94.50 | 97.00 | 91.85 | 97.16 | 0.8913 |

## Comparison of different feature coding techniques

S Table. Prediction effectiveness of feature coding techniques based on the SVHEHS descriptor with raw information on *H. pylori*.

| Feature coding technique | ACC(%) | PRE(%) | SE(%) | SP(%) | MCC |
| --- | --- | --- | --- | --- | --- |
| PseAAC | 87.59 | 87.54 | 87.65 | 87.52 | 0.7520 |
| PseAAC (raw) | 86.97 | 86.70 | 87.38 | 86.56 | 0.7397 |
| AD | 89.16 | 89.46 | 88.82 | 89.51 | 0.7834 |
| AD (raw) | 88.20 | 88.65 | 87.65 | 88.75 | 0.7642 |
| AC | 87.48 | 88.28 | 86.49 | 88.48 | 0.7501 |
| AC (raw) | 86.21 | 86.28 | 86.21 | 86.21 | 0.7247 |

S Table. Prediction effectiveness of feature coding techniques based on the SVHEHS descriptor with raw information on *S. cerevisiae*.

| Feature coding technique | ACC(%) | PRE(%) | SE(%) | SP(%) | MCC |
| --- | --- | --- | --- | --- | --- |
| PseAAC | 93.54 | 95.86 | 91.01 | 96.07 | 0.8719 |
| PseAAC (raw) | 93.44 | 95.71 | 90.95 | 95.92 | 0.8699 |
| AD | 94.37 | 96.90 | 91.67 | 97.07 | 0.8887 |
| AD (raw) | 93.50 | 96.06 | 90.72 | 96.28 | 0.8714 |
| AC | 94.34 | 97.06 | 91.46 | 97.23 | 0.8884 |
| AC (raw) | 93.49 | 96.50 | 90.26 | 96.73 | 0.8717 |

S Table. Prediction effectiveness of feature coding techniques with multi-information fusion and single-feature information on *H. pylori*.

| Feature coding technique | ACC(%) | PRE(%) | SE(%) | SP(%) | MCC |
| --- | --- | --- | --- | --- | --- |
| PseAAC | 87.59 | 87.54 | 87.65 | 87.52 | 0.7520 |
| AD | 89.16 | 89.46 | 88.82 | 89.51 | 0.7834 |
| AC | 87.48 | 88.28 | 86.49 | 88.48 | 0.7501 |
| CT | 86.66 | 86.48 | 86.90 | 86.42 | 0.7332 |
| LD | 88.00 | 86.89 | 89.50 | 86.49 | 0.7606 |
| MMI | 86.25 | 85.94 | 86.69 | 85.80 | 0.7251 |
| ALL(BiGRU) | 96.47 | 96.38 | 96.57 | 96.37 | 0.9294 |


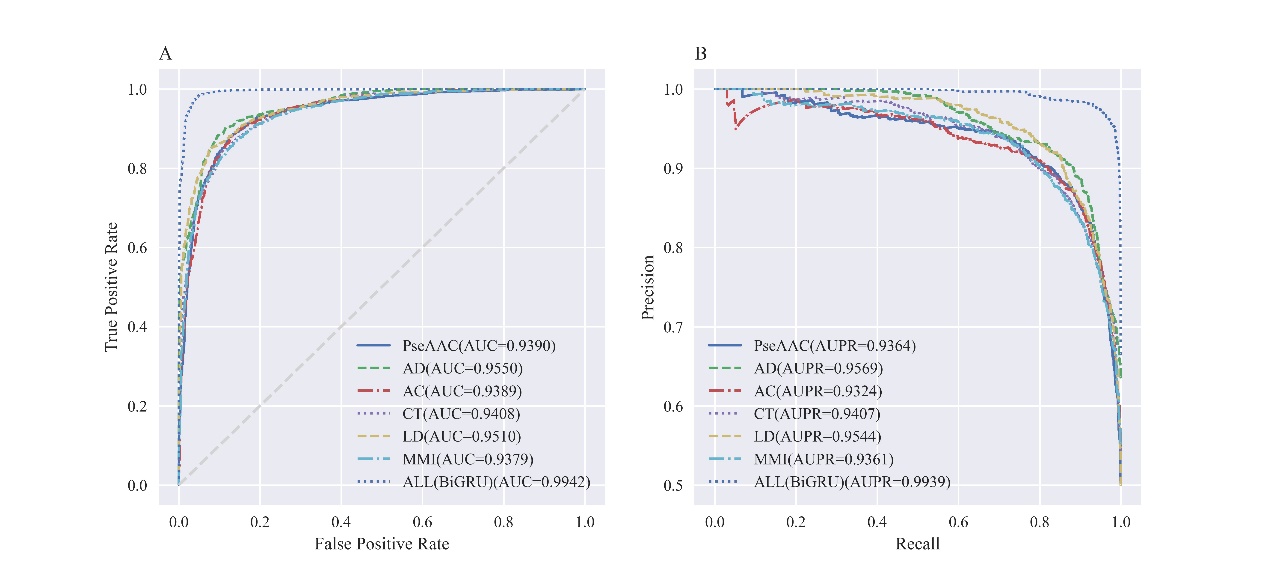


S Fig. Comparison of ROC and PR curves for *H. pylori* between multi-information fusion and single-feature information in feature coding techniques.

## Comparison of different integration methods

S Table. Prediction effectiveness of different integration methods on *H. pylori*.

| Integration method | ACC(%) | PRE(%) | SE(%) | SP(%) | MCC |
| --- | --- | --- | --- | --- | --- |
| MultiCon | 86.97 | 86.25 | 88.00 | 85.94 | 0.7397 |
| MultiSep | 89.92 | 91.35 | 88.20 | 91.63 | 0.7991 |
| MultiEns | 96.47 | 96.38 | 96.57 | 96.37 | 0.9294 |


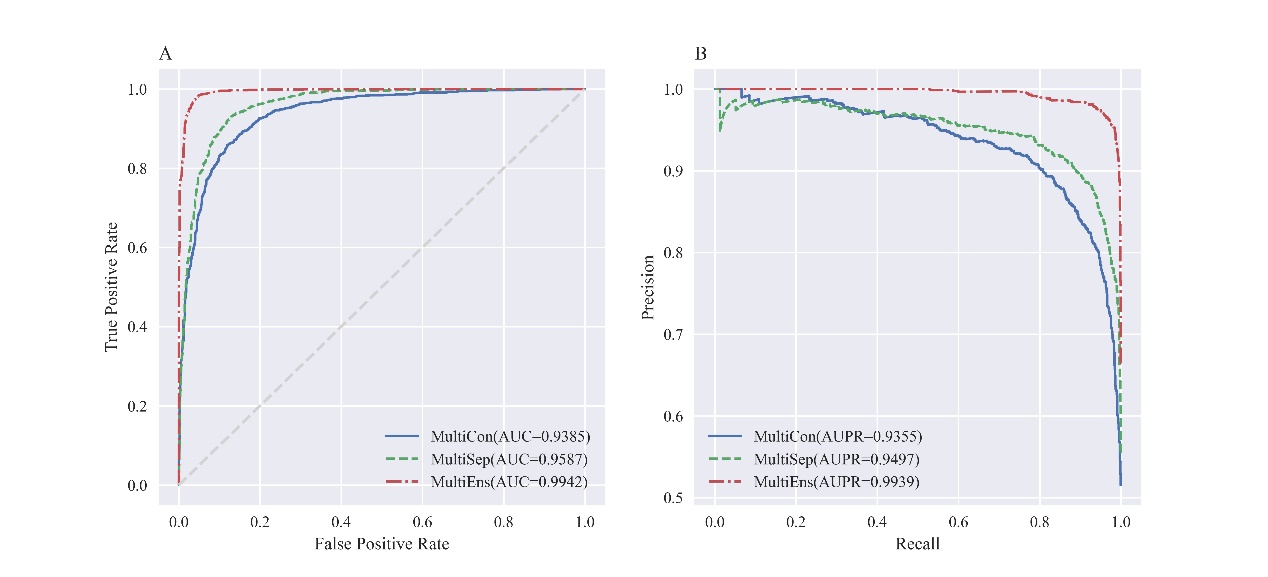


S Fig. Comparison of ROC and PR curves of different integration methods on *H. pylori*.

## Comparison of different directions of GRUs

S Table. Prediction effectiveness of GRU in different directions on *H. pylori*.

| Coding direction | ACC(%) | PRE(%) | SE(%) | SP(%) | MCC |
| --- | --- | --- | --- | --- | --- |
| Forward GRU | 89.23 | 89.50 | 88.89 | 89.57 | 0.7848 |
| Backward GRU | 96.30 | 95.81 | 96.85 | 95.75 | 0.9261 |
| Bidirectional GRU | 96.47 | 96.38 | 96.57 | 96.37 | 0.9647 |


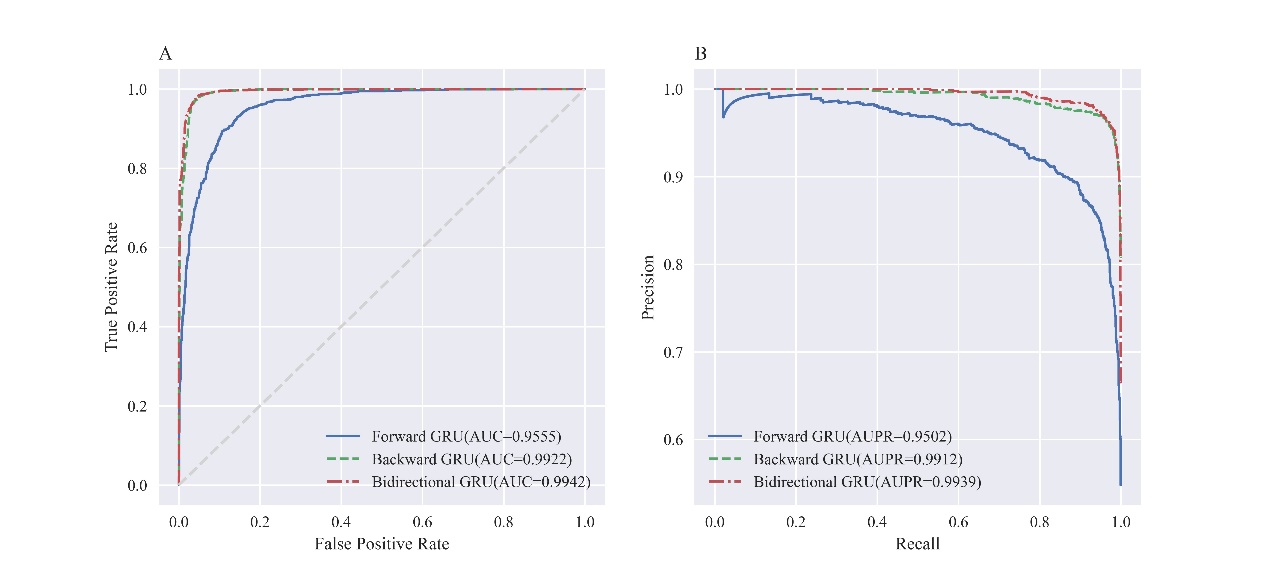


S Fig. Comparison of ROC and PR curves of different directions of GRU on *H. pylori*.

## Classification Results Using the BiGRU

S Table. The number of correctly classified pairs predicted directly by the BiBRU.

| Algorithm | *H.pylori* | *S.cerevisiae* |
| --- | --- | --- |
| Based on PseAAC | 1458 | 5594 |
| Based on AD | 2146 | 6763 |
| Based on AC | 1946 | 6844 |
| Based on CT | 1680 | 6698 |
| Based on LD | 1565 | 6076 |
| Based on MMI | 1529 | 6496 |
| Summary of the most votes | 1674 | 6883 |

## Comparison with traditional classifiers

S Table. Prediction effectiveness of different classifiers on *H. pylori*.

| classifier | ACC(%) | PRE(%) | SE(%) | SP(%) | MCC |
| --- | --- | --- | --- | --- | --- |
| GNB | 70.68 | 66.26 | 84.29 | 57.06 | 0.4299 |
| KNN | 87.79 | 81.81 | 97.26 | 78.32 | 0.7699 |
| SVM | 75.41 | 75.41 | 75.44 | 75.38 | 0.5085 |
| RF | 92.52 | 93.55 | 91.36 | 93.69 | 0.8508 |
| LR | 74.52 | 74.23 | 75.17 | 73.87 | 0.4906 |
| AdaBoost | 82.24 | 81.88 | 82.85 | 81.62 | 0.6449 |
| XGBoost | 96.36 | 95.68 | 97.12 | 95.61 | 0.9275 |
| Extra-Trees | 91.19 | 92.52 | 89.64 | 92.73 | 0.8243 |
| LightGBM | 96.47 | 96.38 | 96.57 | 96.37 | 0.9294 |

S Table. Prediction effectiveness of different classifiers on *S. cerevisiae*.

| classifier | ACC(%) | PRE(%) | SE(%) | SP(%) | MCC |
| --- | --- | --- | --- | --- | --- |
| GNB | 61.57 | 61.26 | 62.98 | 60.15 | 0.2315 |
| KNN | 87.60 | 83.73 | 93.35 | 81.86 | 0.7571 |
| SVM | 59.55 | 58.79 | 64.03 | 55.08 | 0.1920 |
| RF | 96.47 | 96.21 | 96.75 | 96.19 | 0.9294 |
| LR | 64.27 | 63.31 | 67.98 | 60.55 | 0.2863 |
| AdaBoost | 71.61 | 71.96 | 70.88 | 72.34 | 0.4325 |
| XGBoost | 98.34 | 98.43 | 98.25 | 98.43 | 0.9668 |
| Extra-Trees | 96.18 | 96.33 | 96.03 | 96.34 | 0.9237 |
| LightGBM | 97.79 | 97.74 | 97.85 | 97.73 | 0.9559 |


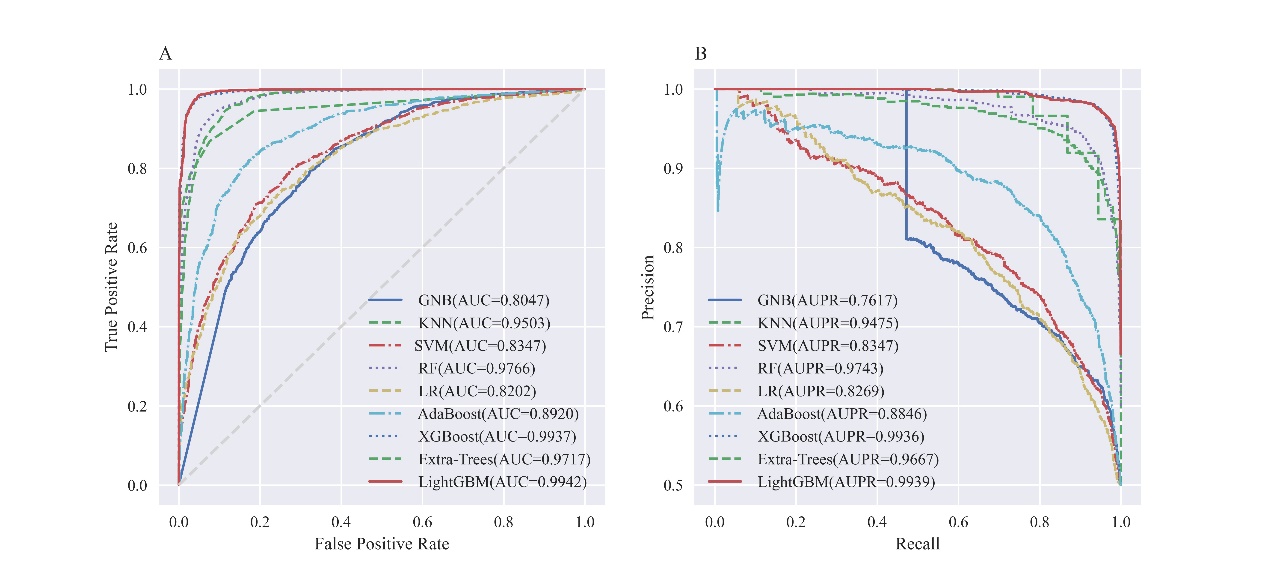


S Fig. Comparison of ROC and PR curves of different classifiers on *H. pylori*.


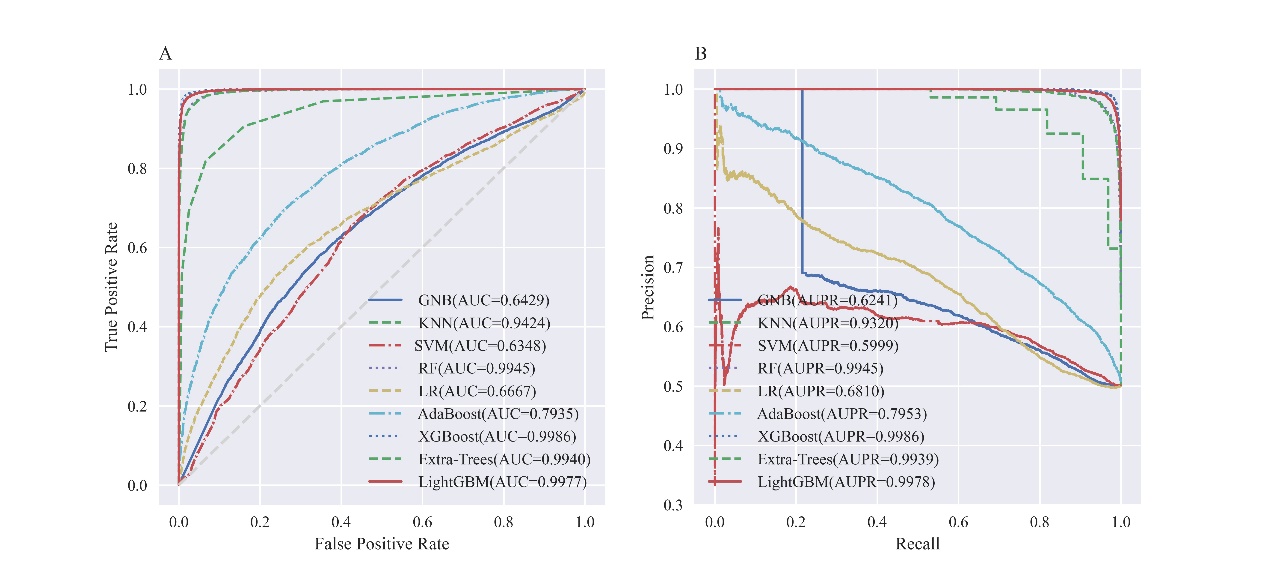


S Fig. Comparison of ROC and PR curves of different classifiers on *S. cerevisiae*.


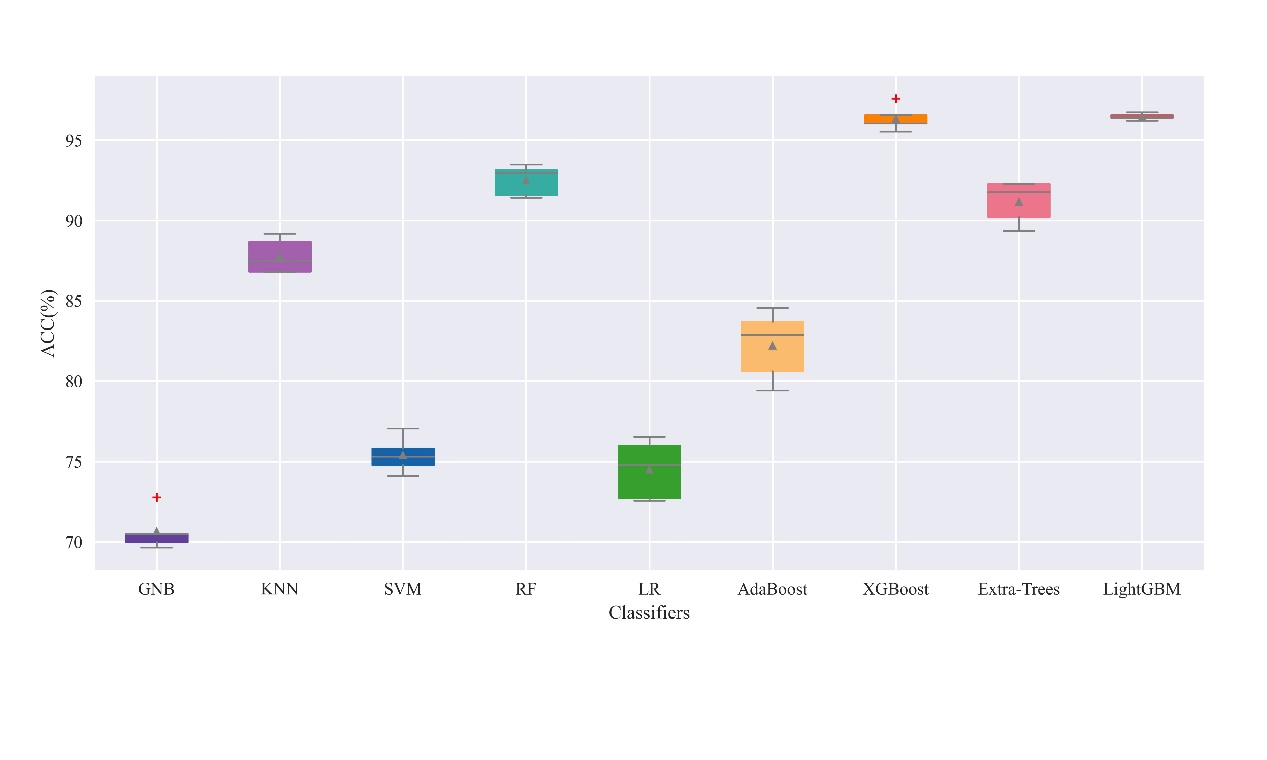


S Fig. Comparison of accuracy of different classifiers on *H. pylori*.


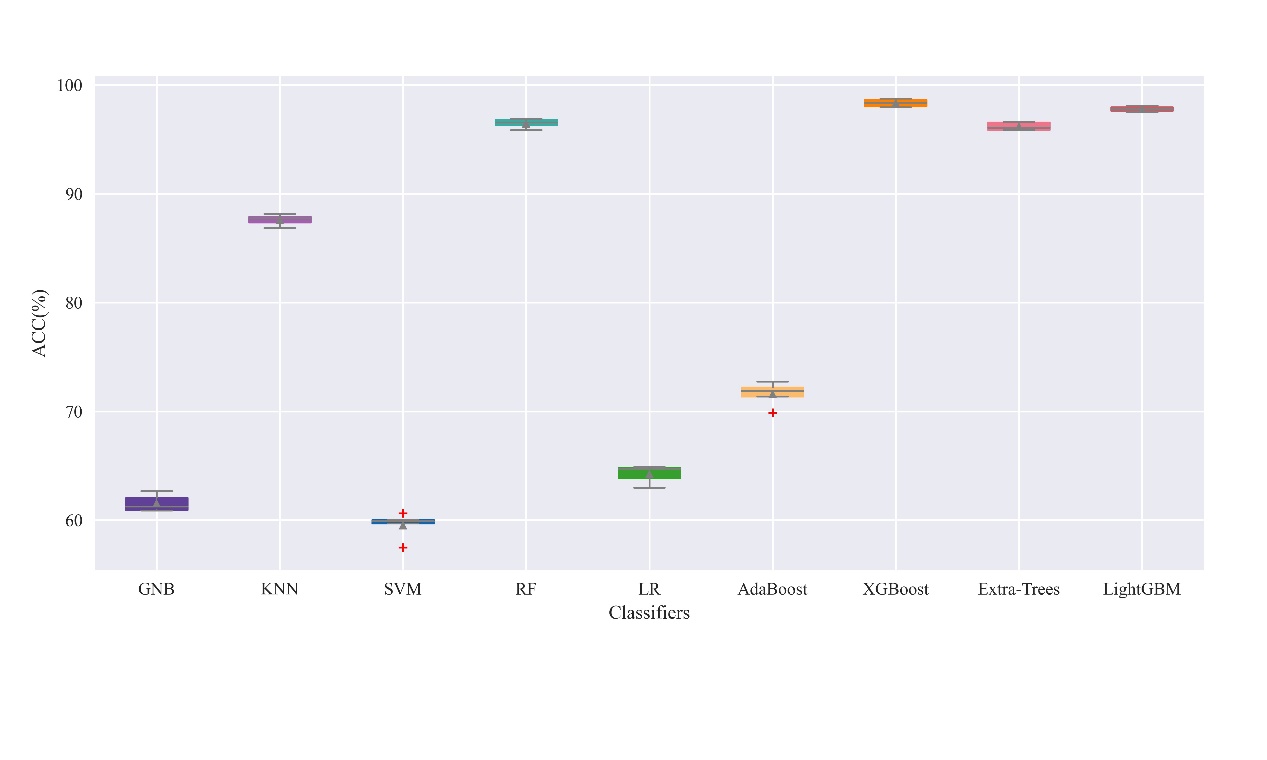


S Fig. Comparison of accuracy of different classifiers on *S. cerevisiae*.

S Table. Running time of different classifiers (in seconds).

| Classifiers | *H. pylori* | *S. cerevisiae* |
| --- | --- | --- |
| GNB | 0.2151 | 0.7736 |
| KNN | 3.5543 | 43.6885 |
| SVM | 73.0581 | 1478.3565 |
| RF | 12.8354 | 61.7091 |
| LR | 0.5934 | 1.7174 |
| AdaBoost | 30.0994 | 122.6098 |
| XGBoost | 9.7112 | 16.5003 |
| Extra-Trees | 3.9094 | 19.1058 |
| LightGBM | 3.9291 | 5.8671 |

(Note: Experiments are conducted on a Windows 10 64-bit system with an Intel(R) Xeon(R) Gold 5218R CPU @ 2.10GHz 2.10GHz (2 processors) processor.)

## Comparison with different advanced models

S Table. Prediction effectiveness of different advanced models on *H. pylori*.

| Model | ACC(%) | PRE(%) | SE(%) | MCC |
| --- | --- | --- | --- | --- |
| LPP+RF[5] | 92.56±0.86 | 94.11±0.99 | 90.82±0.93 | 0.8622±0.0147 |
| MatPCA+WSRC[6] | 83.64±1.15 | 89.71±0.79 | 75.98±1.63 | 0.7226±0.0149 |
| GTB-PPI[7] | 90.47±0.84 | 89.99±2.06 | 91.15±1.42 | 0.8100±0.0163 |
| Gabor+RF[8] | 86.45±0.90 | 88.51±0.82 | 83.82±1.55 | 0.7653±0.0130 |
| NVDT[9] | 94.63±2.18 | 97.56±1.64 | 91.56±4.02 | 0.8949±0.0419 |
| LightGBM-PPI[10] | 89.03 | 88.36 | 89.99 | 0.7814 |
| PCVMZM[11] | 91.25±1.1 | 90.06±2.4 | 92.05±0.6 | 0.8404±0.017 |
| PsePSSM+RF[12] | 89.75±1.67 | 90.18±2.74 | 89.12±1.83 | 0.8162±0.0269 |
| GcForest-PPI[13] | 89.26±1.07 | 88.95±1.36 | 89.71±2.26 | 0.7857±0.0212 |
| Our model | 96.47±0.17 | 96.38±0.55 | 96.57±0.72 | 0.9294±0.0034 |

S Table. Prediction effectiveness of different advanced models on *S. cerevisiae*.

| Model | ACC(%) | PRE(%) | SE(%) | MCC |
| --- | --- | --- | --- | --- |
| LPP+RF[5] | 92.81±0.66 | 96.80±0.68 | 88.55±0.95 | 0.8661±0.0115 |
| MatPCA+WSRC[6] | 94.55±0.63 | 92.33±0.91 | 97.15±0.42 | 0.8968±0.0112 |
| GTB-PPI[7] | 95.15±0.25 | 97.97±0.60 | 92.21±0.36 | 0.9045±0.0053 |
| Gabor+RF[8] | 92.10±0.29 | 93.85±0.69 | 90.09±0.86 | 0.8543±0.0049 |
| PIPR[2] | 97.09±0.24 | 97.00±0.65 | 97.17±0.44 | 0.9417±0.0048 |
| DF-PPI[14] | 96.34±0.34 | 97.56±0.39 | 95.05±0.58 | 0.9270±0.0067 |
| DeepCF-PPI[15] | 95.6±0.57 | 93.4±0.69 | 97.81±0.54 | 0.914±0.0113 |
| GcForest-PPI[13] | 95.44±0.18 | 98.05±0.25 | 92.72±0.44 | 0.9102±0.0035 |
| ProtXLNet[16] | 94.79±0.42 | 97.59±0.53 | 91.88±0.61 | 0.8975±0.0084 |
| Our model | 97.79±0.22 | 97.74±0.41 | 97.85±0.48 | 0.9559±0.0044 |

S Table. Prediction effectiveness on *H. pylori*.

| Model | ACC(%) | PRE(%) | SE(%) | MCC |
| --- | --- | --- | --- | --- |
| LightGBM-PPI(original) | 89.03 | 88.36 | 89.99 | 0.7814 |
| LightGBM-PPI(reproduced) | 82.41±3.78 | 95.22±1.56 | 68.18±7.03 | 0.6767±0.0660 |
| NVDT(original) | 94.63±2.18 | 97.56±1.64 | 91.56±4.02 | 0.8949±0.0419 |
| NVDT(reproduced) | 96.05±2.31 | 98.85±0.67 | 93.17±4.20 | 0.9231±0.0441 |
| Our model | 96.47±0.17 | 96.38±0.55 | 96.57±0.72 | 0.9294±0.0034 |

S Table. Prediction effectiveness on *S. cerevisiae*.

| Model | ACC(%) | PRE(%) | SE(%) | MCC |
| --- | --- | --- | --- | --- |
| LightGBM-PPI(original) | 95.07 | 97.82 | 92.21 | 0.9030 |
| LightGBM-PPI(reproduced) | 97.59±1.83 | 98.72±1.91 | 96.48±3.69 | 0.9529±0.0351 |
| NVDT(original) | 98.28±0.33 | 98.87±0.47 | 97.68±0.33 | 0.9658±0.0067 |
| NVDT(reproduced) | 98.31±0.24 | 98.98±0.29 | 97.62±0.23 | 0.9662±0.0048 |
| Our model | 97.79±0.22 | 97.74±0.41 | 97.85±0.48 | 0.9559±0.0044 |

## Comparison of test results across two training datasets

S Table. A comparison of the prediction results from models trained on the *H. pylori* dataset and the *S. cerevisiae* dataset, respectively, using the first type of test set.

| Training dataset | Test dataset | ACC(%) | SE(%) | F1(%) |
| --- | --- | --- | --- | --- |
| *H.pylori* | *C.elegans* | 45.75 | 45.75 | 62.78 |
|  | *E.coli* | 53.42 | 53.42 | 69.64 |
|  | *H.sapiens* | 42.92 | 42.92 | 60.06 |
|  | *M.musculus* | 40.58 | 40.58 | 57.73 |
| *S.cerevisiae* | *C.elegans* | 94.14 | 94.14 | 96.98 |
|  | *E.coli* | 96.89 | 96.89 | 98.42 |
|  | *H.sapiens* | 95.96 | 95.96 | 97.94 |
|  | *M.musculus* | 94.89 | 94.89 | 97.38 |

S Table. A comparison of the prediction results from models trained on the *H. pylori* dataset and the *S. cerevisiae* dataset, respectively, using the second type of test set.

| Training dataset | Test dataset | ACC(%) | SE(%) | F1(%) |
| --- | --- | --- | --- | --- |
| *H.pylori* | *Disease-specific* | 75.00 | 75.00 | 85.71 |
|  | *One-core network* | 50.00 | 50.00 | 66.67 |
|  | *Wnt-related pathway* | 70.83 | 70.83 | 82.93 |
| *S.cerevisiae* | *Disease-specific* | 98.15 | 98.15 | 99.07 |
|  | *One-core network* | 100.00 | 100.00 | 100.00 |
|  | *Wnt-related pathway* | 100.00 | 100.00 | 100.00 |

**References**

1. Cho K, Van Merrienboer B, Gulcehre C, Bahdanau D, Bougares F, Schwenk H, et al. Learning Phrase Representations using RNN Encoder-Decoder for Statistical Machine Translation. In: Proceedings of conference on empirical methods in natural language processing. Doha, Qatar: ACL; 2014. p. 1724-1734.
2. Chen M, Ju CJT, Zhou G, Chen X, Zhang T, Chang KW, et al. Multifaceted protein-protein interaction prediction based on Siamese residual RCNN. Bioinformatics. 2019;35(14):I305-I314.
3. Ke G, Meng Q, Finley T, Wang T, Chen W, Ma W, et al. LightGBM: A Highly Efficient Gradient Boosting Decision Tree. In: Proceedings of the 31st International Conference on Neural Information Processing Systems. Red Hook, NY, USA: Curran Associates Inc.; 2017. p. 3149-3157.
4. Chen T, Guestrin C. XGBoost: A Scalable Tree Boosting System. In: Proceedings of the 22nd ACM SIGKDD International Conference on Knowledge Discovery and Data Mining. New York, NY, USA: Association for Computing Machinery; 2016. p. 785-794.
5. Zhan X, Xiao M, You Z, Yan C, Guo J, Wang L, et al. Predicting Protein-Protein Interactions Based on Ensemble Learning-Based Model from Protein Sequence. Biology. 2022;11(7):995.
6. Wang Z, Li Y, You ZH, Li LP, Zhan XK, Pan J. Prediction of Protein-Protein Interactions from Protein Sequences by Combining MatPCA Feature Extraction Algorithms and Weighted Sparse Representation Models. Mathematical Problems in Engineering. 2020;2020:5764060.
7. Yu B, Chen C, Zhou H, Liu B, Ma Q. GTB-PPI: Predict Protein-protein Interactions Based on L1-regularized Logistic Regression and Gradient Tree Boosting. Genomics Proteomics & Bioinformatics. 2020;18(5):582-592.
8. Zhan XK, You ZH, Li LP, Li Y, Wang Z, Pan J. Using Random Forest Model Combined With Gabor Feature to Predict Protein-Protein Interaction From Protein Sequence. Evolutionary Bioinformatics. 2020;16:1176934320934498.
9. Zhao N, Zhuo M, Tian K, Gong X. Protein-protein interaction and non-interaction predictions using gene sequence natural vector. Communications Biology. 2022;5(1):652.
10. Chen C, Zhang Q, Ma Q, Yu B. LightGBM-PPI: Predicting protein-protein interactions through LightGBM with multi-information fusion. Chemometrics and Intelligent Laboratory Systems. 2019;191:54-64.
11. Wang Y, You Z, Li X, Chen X, Jiang T, Zhang J. PCVMZM: Using the Probabilistic Classification Vector Machines Model Combined with a Zernike Moments Descriptor to Predict Protein-Protein Interactions from Protein Sequences. International Journal of Molecular Sciences. 2017;18(5):1029.
12. Wang L, You ZH, Chen X, Li JQ, Yan X, Zhang W, et al. An ensemble approach for large-scale identification of protein-protein interactions using the alignments of multiple sequences. Oncotarget. 2017;8(3):5149-5159.
13. Yu B, Chen C, Wang X, Yu Z, Ma A, Liu B. Prediction of protein-protein interactions based on elastic net and deep forest. Expert Systems with Applications. 2021;176:114876.
14. Tran HN, Nguyen PXQ, Guo F, Wang J. Prediction of Protein-Protein Interactions Based on Integrating Deep Learning and Feature Fusion. International Journal of Molecular Sciences. 2024;25(11):5820.
15. Tran HN, Xuan QNP, Nguyen TT. DeepCF-PPI: improved prediction of protein-protein interactions by combining learned and handcrafted features based on attention mechanisms. Applied Intelligence. 2023;53(14):17887-17902.
16. Kimothi D, Biyani P, Hogan JM, Davis MJ. Sequence Representations and Their Utility for Predicting Protein-Protein Interactions. IEEE-ACM Transactions on Computational Biology and Bioinformatics. 2023;20(1):646-657.
